# The identification of mediating effects using genome-based restricted maximum likelihood estimation

**DOI:** 10.1371/journal.pgen.1010638

**Published:** 2023-02-21

**Authors:** Cornelius A. Rietveld, Ronald de Vlaming, Eric A. W. Slob

**Affiliations:** 1 Department of Applied Economics, Erasmus School of Economics, Erasmus University Rotterdam, Rotterdam, The Netherlands; 2 Erasmus University Rotterdam Institute for Behavior and Biology, Erasmus School of Economics, Erasmus University Rotterdam, Rotterdam, The Netherlands; 3 Department of Economics, School of Business and Economics, Vrije Universiteit Amsterdam, Amsterdam, The Netherlands; 4 Medical Research Council Biostatistics Unit, School of Clinical Medicine, University of Cambridge, Cambridge, United Kingdom; University Hospital of the Canton Vaud (CHUV), SWITZERLAND

## Abstract

Mediation analysis is commonly used to identify mechanisms and intermediate factors between causes and outcomes. Studies drawing on polygenic scores (PGSs) can readily employ traditional regression-based procedures to assess whether trait *M* mediates the relationship between the genetic component of outcome *Y* and outcome *Y* itself. However, this approach suffers from attenuation bias, as PGSs capture only a (small) part of the genetic variance of a given trait. To overcome this limitation, we developed MA-GREML: a method for Mediation Analysis using Genome-based Restricted Maximum Likelihood (GREML) estimation.

Using MA-GREML to assess mediation between genetic factors and traits comes with two main advantages. First, we circumvent the limited predictive accuracy of PGSs that regression-based mediation approaches suffer from. Second, compared to methods employing summary statistics from genome-wide association studies, the individual-level data approach of GREML allows to directly control for confounders of the association between *M* and *Y*. In addition to typical GREML parameters (e.g., the genetic correlation), MA-GREML estimates (*i*) the effect of *M* on *Y*, (*ii*) the *direct effect* (i.e., the genetic variance of *Y* that is not mediated by *M*), and (*iii*) the *indirect effect* (i.e., the genetic variance of *Y* that is mediated by *M*). MA-GREML also provides standard errors of these estimates and assesses the significance of the indirect effect.

We use analytical derivations and simulations to show the validity of our approach under two main assumptions, *viz*., that *M* precedes *Y* and that environmental confounders of the association between *M* and *Y* are controlled for. We conclude that MA-GREML is an appropriate tool to assess the mediating role of trait *M* in the relationship between the genetic component of *Y* and outcome *Y*. Using data from the US Health and Retirement Study, we provide evidence that genetic effects on Body Mass Index (BMI), cognitive functioning and self-reported health in later life run partially through educational attainment. For mental health, we do not find significant evidence for an indirect effect through educational attainment. Further analyses show that the additive genetic factors of these four outcomes do partially (cognition and mental health) and fully (BMI and self-reported health) run through an earlier realization of these traits.

## Author summary

Mediation analysis is instrumental to identify intermediate factors between causes and outcomes. We developed Mediation Analysis using Genome-based Restricted Maximum Likelihood (MA-GREML) estimation to quantify to which degree the genetic component (*cause*) of a trait (*outcome*) is mediated by another trait (*intermediate factor*). This method has two main advantages. First, it captures the total additive genetic variance as estimated using GREML rather than the smaller part of the genetic variance typically captured by polygenic scores. Second, the individual-level data approach GREML takes allows users to directly control for confounders in the model. We use analytical derivations, simulations, and empirical data to validate the underlying model that is used to quantify to which degree an intermediate trait mediates the relationship between the genetic component of the outcome and the outcome itself. We conclude that MA-GREML is an appropriate method to estimate genetic mediation and to test the significance of the indirect effect. We implemented the estimation procedure in the freely available Python-based software package MGREML, available at https://github.com/devlaming/mgreml/.

## Introduction

Mediation analysis is widely used to identify the mechanisms and intermediate factors in between causes and outcomes [1]. The original idea of mediation analysis dates back to at least as early as 1934 [2], but it gained momentum after its seminal introduction in the social sciences by Baron and Kenny almost 40 years ago now [3]. Since then, the number of studies using mediation analysis has grown exponentially [4].

Motivated by the desire to control for genetic confounding [5], the earlier twin-study literature already attempted to investigate the effect of an environmental factor on a trait while controlling for genetic influences assuming that a significant relationship between the mediating variable and the outcome variable in a genetically-informative context would provide evidence for mediation [6]. This approach is not guaranteed to be unbiased because the shared environment (family) component in a twin study may pick up parental genetic influences; moreover, this approach fails in case the mediating variable is shared between twins [6]. The more recently developed biometric mediation model for twin data [7] can detect genetic confounding in a mediation setting, but has been designed to assess mediation between observed (non-genetic) variables in a genetically-informative model.

The Baron and Kenny approach, though, to assess mediation draws upon conventional regression techniques in a sample of unrelated individuals. By establishing that the effect of the independent variable on the outcome variable significantly changes upon inclusion of the mediating variable in the model, one can assess mediation [3, 8]. By drawing on polygenic scores (PGSs), several recent studies have employed such regression models to identify the mechanisms through which genotypes affect traits [9–11]. A clear limitation of this approach is that PGSs have limited explanatory power and as such do not fully account for the additive genetic component of traits [12–15]. Although several regression approaches have been developed recently to ‘disattenuate’ the estimated effect of the PGS from measurement error [15–17], these approaches cannot be used to properly account for the total additive genetic component of the outcome variable in a mediation model because these approaches do not recover the relationship between the main explanatory variable (the PGS) and the mediating variable. To overcome this limitation of PGS-based mediation analyses, we developed a structural equation model (SEM) for mediation analysis using Genome-based Restricted Maximum Likelihood (GREML) estimation. This approach we refer to as MA-GREML and has been implemented in MGREML, a Python-based command-line tool [18, 19].

Originally, GREML estimation has been developed to quantify the contribution of random, additive effects of single-nucleotide polymorphisms (SNPs) to variation in a trait [20, 21] (i.e., the genetic variance) and to the covariance between traits [22] (i.e., the genetic covariance). Moreover, GREML also quantifies the environmental variance of traits (i.e., the phenotypic variance not accounted for by the random SNP effects) and the environmental covariance between traits (i.e., the phenotypic covariance not accounted for by the SNP effects). Therefore, MA-GREML is able to quantify not only the genetic (co)variance of supposed mediator *M* and outcome *Y*, but also the genetic variance of *Y* that is mediated by *M* (i.e., the *indirect effect*) and the genetic variance of *Y* that is not mediated by *M* (i.e., the *direct effect*). Importantly, these estimates are not subject to the typical attenuation bias one would expect in a mediation analysis using a noisy approximation of the genetic component of *M* and/or *Y*, *viz*., a PGS.

MA-GREML, like Genomic SEM [23], relies on a structural equation model (SEM). Importantly, whereas MA-GREML employs individual-level genetic data Genomic SEM draws upon genome-wide association study (GWAS) summary statistics. Genomic SEM is a flexible two-stage tool that allows users to specify and estimate a wide range of SEMs, also ones involving forms of mediation. Nevertheless, MA-GREML uses a specific SEM focused on the question to which degree the relationship between the genetic component of outcome *Y* and outcome *Y* itself is mediated by mediator *M*. Genomic SEM cannot be utilized to answer this specific question because such a model based on GWAS summary statistics for *M* and *Y* would involve more parameters than there are degrees of freedom in the model. We refer to Section F in S1 Text for a more elaborate discussion of mediation analysis using Genomic SEM.

Mendelian randomization (MR) models, in which the main explanatory variable is only allowed to impact the outcome variable through the mediator, can be conceptualized as a restricted version of the MA-GREML model. This restriction boils down to the so-called *exclusion assumption* to hold in an MR study, which is often debatable and not empirically verifiable [24, 25]. MA-GREML may however be instrumental to provide evidence in favor of the exclusion restriction to hold (i.e., whether there is indeed full mediation). Relatedly, a GWAS is sometimes conducted on a proxy-trait that is easier to measure than the trait of interest itself [26]. Evidence that genetic effects on the proxy trait run largely or even fully through the actual trait of interest may build confidence in such proxy-GWAS findings.

We use analytical derivations, simulations, and empirical data, to validate the structural model underlying MA-GREML and the procedure developed to test the significance of mediating effects. For the empirical analyses, we draw on longitudinal data from the US Health and Retirement Study [27]. While we do find evidence that genetic effects on Body Mass Index (BMI), cognition and self-reported health in later life run through educational attainment, the evidence regarding the mediating role of educational attainment for mental health in later life is inconclusive. In addition, we find that the additive genetic factors of these four later-life health outcomes do partially (cognition and mental health) and fully (BMI and self-reported health) run through an earlier realization of these traits.

## Description of the method

In this section, we first revisit univariate and bivariate GREML estimation. Second, we present our main SEM, its underlying assumptions, and the interpretation of the latent factors in the model. Third, we formulate the main quantities of interest (e.g., the *direct effect* and *indirect effect*) as functions of the genetic and environmental (co)variance of *M* and *Y*. Fourth, we propose a simple likelihood-ratio test (LRT) to assess whether the indirect effect differs from zero. Finally, we compare MA-GREML estimation to a step-wise approach involving several univariate GREML analyses that resembles the procedure suggested by Baron and Kenny [3] to test for mediation in a regression framework.

### GREML estimation

The default form of GREML estimation considers a single trait *Y*, for which it assumes fixed effects (i.e., non-random) of the confounders and additive, random effects of the SNPs, where each SNP is assumed to have the same explained variance (*R*^2^) with respect to the trait of interest [20, 21]. Other specifications of how the *R*^2^ per SNP may vary across the genome are possible. Such a specification is referred to as a heritability model [28]. Typically, this specification only affects the way in which the so-called genomic relatedness matrix (GRM) is calculated [18, 29].

Under this model, *Y* can effectively be decomposed into (*i*) a latent genetic factor *G*_*Y*_, (*ii*) a latent environmental factor *E*_*Y*_, and (*iii*) a contribution from the fixed-effect confounders. This decomposition can be perceived as a simple SEM, involving two latent factors, *viz*., *G*_*Y*_ and *E*_*Y*_. The covariance of a given genetic factor between two individuals is equal to the corresponding element from the GRM, which reflects genetic similarity between individuals based on SNP data. Thus, the more genetically similar two individuals are, the more similar they tend to be in terms of *G*_*Y*_ (i.e., their genetic predisposition towards *Y*). The covariance of a given environmental factor is assumed to be zero across individuals: environmental exposures (other than those captured by the fixed-effect confounders) are assumed to be uncorrelated across individuals. Without loss of generality, we also assume that environmental factors have unit variance and that all latent factors have an expectation equal to zero.

Omitting fixed effects in all equations for presentation purposes, this simple SEM can be written as:

$$\begin{array}{c} Y=\alpha G_{Y}+\beta E_{Y}, \end{array}$$ (1)

where $\sigma_{YY}^{G}=\alpha^{2}$ is the variance in *Y* accounted for by *G*_*Y*_ (i.e., the *genetic variance* of *Y*) and $\sigma_{YY}^{E}=\beta^{2}$ the variance accounted for by *E*_*Y*_ (i.e., the *environmental variance* of *Y*). Quantities $\sigma_{YY}^{G}$ and $\sigma_{YY}^{E}$ are so-called variance components (VCs) [30]. Univariate GREML applied to *Y* provides estimates of these VCs while controlling for user-specified fixed-effect confounders. In this framework, the SNP-based heritability ($h_{\text{SNPs}}^{2}$) of *Y* is defined as

$$\begin{array}{c} h_{\text{SNPs}}^{2}=\frac{\sigma_{YY}^{G}}{\sigma_{YY}^{G}+\sigma_{YY}^{E}}, \end{array}$$ (2)

reflecting the proportion of variance (in *Y*) accounted for by the additive SNP effects under the specified heritability model.

Bivariate and multivariate generalizations of GREML [18, 22, 31] allow users to also estimate genetic and environmental covariance *between* traits (denoted by $\sigma_{MY}^{G}$ and $\sigma_{MY}^{E}$ respectively, for traits *M* and *Y*). Genetic covariance quantifies the phenotypic covariance between two traits accounted for by random SNP effects under the specified heritability model. Environmental covariance quantifies the remaining phenotypic covariance. These covariances are also referred to as VCs. In this framework, the genetic correlation between *M* and *Y* is defined as

$$\begin{array}{c} \rho_{MY}^{G}=\frac{\sigma_{MY}^{G}}{\sqrt{\sigma_{MM}^{G}\sigma_{YY}^{G}}}. \end{array}$$ (3)

Similar to a classical correlation coefficient, $\rho_{MY}^{G}$ can range between −1 and +1. In case $\rho_{MY}^{G}=\pm 1$, the genetic components of the two traits are perfectly correlated.

A relatively parsimonious SEM for two traits could be a model in which there are two genetic factors, with the first genetic factor only affecting *M* and the second genetic factor affecting both *M* and *Y*. Such a SEM would only be one possible representation of many statistically equivalent models, that can all yield a valid $\rho_{MY}^{G}$ (i.e., in between −1 and +1). Given the number of options for the design of a SEM, its specification should be guided by plausible assumptions.

### Structural equation model

**Fig 1:**
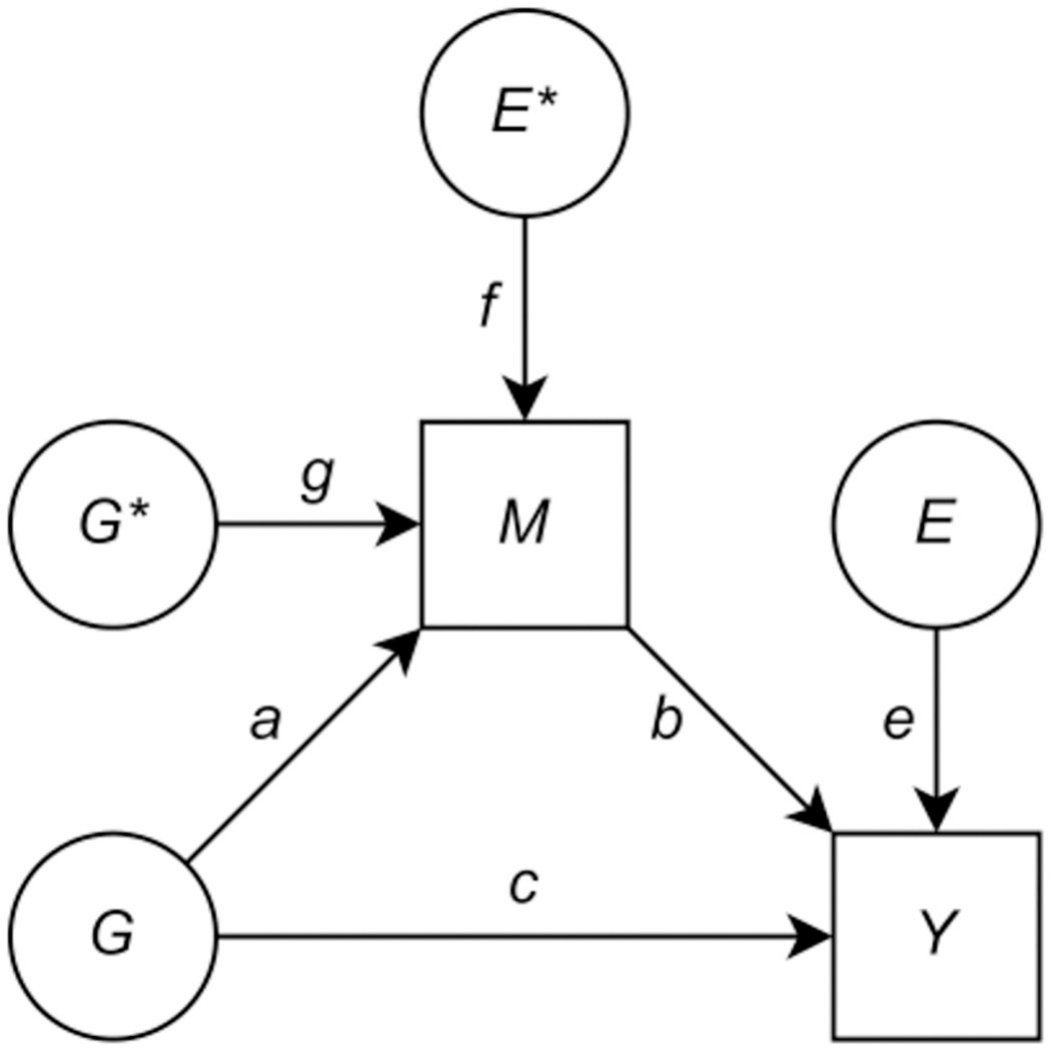
Structural equation model (SEM) for mediation analysis using GREML (MA-GREML), to quantify to which extent the genetic component of trait *Y* affects trait *Y* through trait *M*. *G* and G* are latent genetic factors, *E* and E* are latent environmental factors, *M* is the observed mediator, *Y* is the observed outcome, (*a*^2^ + *g*^2^)*b*^2^ is the genetic variance of *Y* that is mediated by *M* (*indirect effect*), and *c*^2^ is the genetic variance of *Y* that is not mediated by *M* (*direct effect*); *Full mediation*: direct effect = 0 and indirect effect > 0; *Partial mediation*: direct and indirect effect both > 0; *No mediation*: direct effect > 0 and indirect effect = 0.

For the purpose of MA-GREML, we make the following set of assumptions, which yields the SEM shown in Fig 1:

A1 The heritability model has been correctly specified.
A2 Mediator *M* precedes outcome *Y* and has homogeneous effect *b* on *Y*.
A3 The environmental component of *M* influences *Y* only through *M*.
A4 Factors that violate A3 (*environmental confounders*) are controlled for.
A5 *M* has non-zero environmental variance, also when controlling for environmental confounders.

Among these, Assumptions A2 and A4 are most critical. To see their relative importance, observe that Assumption A1 has already been the subject of much research. Importantly, deviations from the assumed heritability model can to a considerable extent be dealt with by modifying the calculation of elements in the GRM [29]. Moreover, although Assumption A3 is rather strict at first glance, some violations are permissible, provided these are due to observed confounders that can be controlled for (see Assumption A4). Finally, Assumption A5 implies the requirement of *M* having $h_{\text{SNPs}}^{2}<1$, which is exceedingly likely in empirical applications, as virtually all human traits are influenced by both genetic and environmental factors [32, 33].

Although Assumption A2 is quite typical in mediation analysis [8], this assumption is hard to verify empirically. Types of mediation analyses using tools such as Genomic SEM [23] also hinge on the causal direction from mediator to outcome being correctly specified. Imposing this assumption requires solid theoretical motivation and argumentation.

Assumption A4 is equivalent to the following two requirements: after controlling for the observed environmental confounders (*i*) there exists no other latent environmental factor that has a direct effect on both *M* and *Y* and (*ii*) what then remains in terms of *E* and E* is uncorrelated. The reason for introducing Assumption A4, as we show in Section C in S1 Text, is that only then the environmental covariance between *M* and *Y* is purely driven by the causal effect of *M* on *Y*. Thus, Assumption A4 allows to consistently estimate *b* using the environmental covariance. We stress that the set of environmental confounders included in the model should not contain colliders, as their inclusion in the model leads to collider bias.

In light of Assumption A4, a notable advantage of GREML is that it uses individual-level data, allowing users to directly control for aforementioned confounders of the association between *M* and *Y* when identified using the environmental covariance. Genomic SEM [23], for instance, relies on GWAS summary statistics. As indicated previously, Genomic SEM is unable to identify the SEM in Fig 1, because it typically has no information about the environmental (co)variance of traits at its disposal, and, thus, has too few degrees of freedom for identification of all relevant parameters. However, even if an extension of Genomic SEM would be able to identify the SEM in Fig 1 by leveraging sample overlap between a GWAS of *M* and a GWAS of *Y* (and estimates of the environmental covariance between *M* and *Y* based on that), and even though it may be convenient to use publicly available GWAS summary statistics from well-powered GWASs for the desired type of mediation analyses, adequately controlling for such confounders of the association between *M* and *Y* remains difficult—in a GWAS one typically only controls for (a rather limited set of) confounders of the association between the SNP and the outcome, and not for confounders of the associations between various traits when identified from the environmental (co)variance permeating the summary statistics.

### Interpretation of the latent factors

In the SEM shown in Fig 1, *G* and G* are latent genetic factors, *E* and E* are latent environmental factors, *M* is the observed mediator, and *Y* is the observed outcome. Although observed environmental confounders are not drawn in this figure (for the sake of clarity of the diagram), these can readily be controlled for by MA-GREML.

Conceptually, *G* is the genetic component of *M* that is also permitted to affect *Y* directly, whereas G* is the genetic component of *M* that is only permitted to have an indirect effect on *Y*, *viz*., through *M*. To understand why both *G* and G* are relevant for this model, first observe that without *G* the model would simply enforce full mediation: in that case there would only be genetic factor G* of which its effect on *Y* runs fully through *M*. Second, without G*, the model would enforce a perfect genetic correlation between *M* and *Y* as both traits are then affected only by shared genetic factor *G* in that case, irrespective of whether there is no mediation, partial mediation, or full mediation. For a more elaborate explanation for why both *G* and G* are needed in the model, we refer to Section D in S1 Text.

The presence of environmental factor E* (i.e., with *f* ≠ 0) is required to satisfy Assumption A5: *M* has $h_{\text{SNPs}}^{2}<1$. Environmental factor *E* permits *Y* to have $h_{\text{SNPs}}^{2}<1$ even if *b* = 0. Here, we emphasize that MA-GREML would still yield valid estimates if applied to a dataset for which in truth *e* = 0 (i.e., *E* plays no role). Thus, *E* merely enhances versatility of MA-GREML. A similar argument could be made in favor of including an idiosyncratic genetic factor *G*′ that only affects *Y*: here MA-GREML also still yields valid results (see also Section E in S1 Text). However, for brevity and clarity of the presentation of our model, we do not explicitly consider the presence of such a factor *G*′ here.

### Direct effect and indirect effect

The underlying equations of the SEM in Fig 1 are given by:

$$M=Ga+G^{*}g+E^{*}f,\;\text{and}$$ (4)

Y=Gc+Mb+Ee. (5)

From Eq 4, it follows that $\sigma_{MM}^{G}=a^{2}+g^{2}$ and $\sigma_{MM}^{E}=f^{2}$. Since *M* has effect *b* on *M*, mediator *M* contributes (*a*^2^ + *g*^2^)*b*^2^ to the genetic variance of *Y* (*indirect effect*). Moreover, as *G* is the only genetic factor that has a direct effect on *Y* (with coefficient *c*), the genetic variance not mediated by *M* is given by *c*^2^ (*direct effect*).

In MA-GREML, the effect of *M* on *Y* (i.e., *b*), the direct effect, and the indirect effect are the main quantities of interest (see also the discussion in Section A in S1 Text). Using these definitions, we can distinguish three forms of genetic mediation, *viz*., (*i*) *full mediation* if the direct effect = 0 and the indirect effect > 0, (*ii*) *partial mediation* if the direct effect > 0 and the indirect effect >0, (*iii*) *no mediation* if the direct effect > 0 and the indirect effect = 0. There is also a degenerate case in which both the direct and indirect effect are equal to zero, implying that outcome *Y* has $h_{\text{SNPs}}^{2}=0$. For such an outcome, the question about mediation through *M* is not relevant.

Substituting *M* on the right-hand side of Eq 5 by the right-hand side of Eq 4 yields:

$$Y=Gc+(Gab+G^{*}gb)+E^{*}fb+Ee$$ (6)

$$=G(ab+c)+G^{*}gb+E^{*}fb+Ee,$$ (7)

where *Gc* is the direct contribution of *G* to *Y* and *Gab* + G*gb is the contribution of *G* and G* running through *M*. This formulation reveals that whenever *abc* ≠ 0, these two contributions are correlated (as both then involve *G*). In that case, the genetic variance of *Y* comprises a cross term equal to 2*abc*, in addition to the direct effect and indirect effect. In other words, the genetic variance of *Y* can and often will differ from the sum of the genetic variance mediated by *M* and the genetic variance not mediated by *M*. This deviation cannot clearly be categorized as either part of the direct or indirect effect. We also abstain from that, and treat it as a cross term.

### Identification strategy

Table 1 shows the VCs as function of the coefficients in the main SEM (Fig 1). These relations are derived in Section B in S1 Text. Although not all coefficients of the SEM are always separately identified, the quantities of interest (i.e., *b*, the direct effect, and the indirect effect) are always identified provided Assumption A5 holds true. For instance, in case of full mediation (*c*^2^ = 0 and (*a*^2^ + *g*^2^)*b*^2^ > 0), the SEM cannot differentiate between contributions from G* and *G*. More specifically, in case of full mediation, *g*^2^ and *a*^2^ are not separately identified. However, *a*^2^ + *g*^2^ still is identified.

**Table 1:** Variance components (VCs) of mediator *M* and outcome *Y* as function of the coefficients in the structural equation model (SEM) shown in Fig 1.

VC	Description	SEM coefficients
$\sigma_{MM}^{G}$	Genetic variance of *M*	= *a*^2^ + *g*^2^
$\sigma_{YY}^{G}$	Genetic variance of *Y*	= (*a*^2^ + *g*^2^) *b*^2^ + *c*^2^ + 2*abc*
$\sigma_{MY}^{G}$	Genetic covariance of *M* and *Y*	= (*a*^2^ + *g*^2^) *b* + *ac*
$\sigma_{MM}^{E}$	Environmental variance of *M*	= *f*^2^
$\sigma_{YY}^{E}$	Environmental variance of *Y*	= *f*^2^*b*^2^ + *e*^2^
$\sigma_{MY}^{E}$	Environmental covariance of *M* and *Y*	= *f*^2^*b*

In Section C in S1 Text, we derive a mapping from VCs to the quantities of interest. This mapping can be summarized as:

$$b=\frac{\sigma_{MY}^{E}}{\sigma_{MM}^{E}},$$ (8)

$$\text{indirect}\;\text{effect}=\sigma_{MM}^{G}(\frac{\sigma_{MY}^{E}}{\sigma_{MM}^{E}}{)}^{2},\text{and}$$ (9)

$$\text{direct}\;\text{effect}=\sigma_{YY}^{G}+\sigma_{MM}^{G}(\frac{\sigma_{MY}^{E}}{\sigma_{MM}^{E}}{)}^{2}-2\sigma_{MY}^{G}\frac{\sigma_{MY}^{E}}{\sigma_{MM}^{E}}.$$ (10)

Since these three quantities can be expressed as simple functions of the VCs and since MGREML can readily calculate the sampling covariance matrix of the VCs [18], the delta method can be applied to obtain standard errors (SEs) of the estimates of *b*, the direct effect, and indirect effect. Derivations of the SEs can be found in Section C.4 in S1 Text. By default, MA-GREML as implemented in MGREML returns these SEs.

### Significance of the indirect effect

Assessing the significance of the indirect effect is currently advocated to be the most appropriate test for the presence of mediation [8]. Given we have an estimator (denoted with hat notation) of the indirect effect and a corresponding SE, we can calculate a Wald test statistic *W* as follows:

$$\begin{array}{c} W=(\frac{\hat{\text{indirect}\;\text{effect}}}{\text{SE}(\hat{\text{indirect}\;\text{effect}})}{)}^{2}. \end{array}$$ (11)

Under the null hypothesis of no indirect effect, *W* should asymptotically follow a *χ*^2^(1) distribution [34]. However, the likelihood function of the bivariate GREML model is a nonlinear function of the variance components (VCs) [18] and the indirect effect in turn is a nonlinear transformations of these estimated VCs. For nonlinear models, a multitude of equivalent transformations of restrictions under the null hypothesis can be conceived such that the Wald test statistic takes on any arbitrary non-negative value [35]. This issue is sometimes referred to as the non-invariance of the Wald test. This major limitation of the Wald test is further compounded by the fact that the null hypothesis of no indirect effect corresponds to values of the VCs that partially lie on the edge of the parameter space, *viz*., $\sigma_{MM}^{G}=0$ if $\sigma_{MY}^{E}\neq 0$.

To test for the significance of the indirect effect, we, therefore, instead use a LRT, which has the invariance property that the Wald test lacks. Since the indirect effect is given by (*a*^2^ + *g*^2^)*b*^2^, it can only be non-zero if two conditions are met: (*i*) *b* ≠ 0 and (*ii*) *a*^2^ + *g*^2^ > 0. Conversely, this implies that the parameter space permitted by the null hypothesis of no indirect effect (P) is the union of the space permitted by *a*^2^ + *g*^2^ = 0 (denoted by $P_{a}$) and the space permitted by *b* = 0 (denoted by $P_{b}$). This union contains the intersection, where both *b* = 0 and *a*^2^ + *g*^2^ = 0, as well as all instances where either of the two equals zero.

To obtain the test statistic, we therefore perform the MA-GREML analysis (*i*) within parameter space $P_{a}$, and (*ii*) within parameter space $P_{b}$. By taking the maximum value of the two resulting log-likelihoods we find the optimum of the log-likelihood function in $P=P_{a}\cup P_{b}$ (i.e., the complete parameter space permitted under the null hypothesis). Comparison of this log-likelihood value to that of the standard bivariate model enables an LRT. The degrees of freedom for this LRT are determined by the number of parameters fixed in the model and, therefore, depends on whether the best fit is found in parameter space $P_{a}$ (two degrees of freedom lost) or $P_{b}$ (one degree of freedom lost).

The power of this LRT thus depends on the power to detect (*i*) *a*^2^ + *g*^2^ > 0 and (*ii*) *b* ≠ 0. While (*i*) concerns the power to detect that *M* has a non-zero heritability, (*ii*) is about the power to detect the environmental covariance between *M* and *Y*. The latter is typically high in empirical applications provided that there is sufficient overlap between the sets of individuals for whom *M* and for whom *Y* is observed). Therefore, the power to detect $h_{\text{SNPs}}^{2}>0$ for *M* serves as a reasonable upper bound for the power to detect the indirect effect. For this purpose, the GCTA power calculator [36] can be used to calculate the statistical power to detect $h_{\text{SNPs}}^{2}$ of *M* for a given sample size. This power calculator can be accessed online at https://shiny.cnsgenomics.com/gctaPower/. For example, the power to detect $h_{\text{SNPs}}^{2}>0$ for *M* with true $h_{\text{SNPs}}^{2}=25\%$ equals 100% power in a sample of *N* = 10, 000. The power remains high at 97.7% in a sample of this size if $h_{\text{SNPs}}^{2}$ is halved to 12.5%. Halving $h_{\text{SNPs}}^{2}$ of *M* again (i.e., $h_{\text{SNPs}}^{2}=6.125\%$), we find that we have only 49.0% power to detect the heritable component of *M* in case *N* = 10, 000.

### Mediation analysis using univariate GREML estimation

MA-GREML takes a bivariate approach in which mediator *M* and outcome *Y* are simultaneously included in the model. Regression-based mediation analysis is traditionally performed using a step-wise procedure in which several univariate models with either *M* or *Y* as dependent variable are analyzed and compared [3]. In Section G in S1 Text, we show that a step-wise procedure involving univariate GREML models will only yield consistent results for the SEM in Fig 1 in case of full mediation (i.e., direct effect *c*^2^ = 0) and/or the genetic variance that directly contributes to *Y* does not also affect *M* (i.e., *a* = 0).

In short, mirroring a main critique on the step-wise regression-based mediation analysis procedure [1], a procedure involving univariate GREML models for *M* and *Y* would provide an estimate of *b* that is confounded by genetic factor *G*, and, in turn, uses this confounded estimate to back out which part of the genetic variance of *Y* is not mediated by *M*. In any other situation than the described extreme scenarios, this leads to inconsistent results.

## Verification and comparison

Using simulations, we analyze whether the MA-GREML estimators are consistent and if the LRT for the presence of an indirect effect behaves appropriately (i.e., no inflated false-positive rate for simulations in which the null hypothesis of no indirect effect holds true). Besides a *Baseline* scenario with partial mediation (*a*^2^ + *g*^2^ = 2, *b* = 1, and *c*^2^ = 1), we also consider three scenarios with no mediation (i.e., indirect effect = 0), *viz*., Scenario (*i*) where *a*^2^ + *g*^2^ = 0, *b* = 1, and *c*^2^ = 1; Scenario (*ii*) where *a*^2^ + *g*^2^ = 2, *b* = 0, and *c*^2^ = 1; Scenario (*iii*) where *a*^2^ + *g*^2^ = 0, *b* = 0, and *c*^2^ = 1. Finally, we consider one scenario with full mediation (i.e., direct effect = 0), *viz*., Scenario (*iv*) where *a*^2^ + *g*^2^ = 2, *b* = 1 and *c*^2^ = 0. Section I in S1 Text provides more details about the set-up of the simulations and Table A-E in S1 Data contains simulation output for each run of the simulation.

The simulation results are based on 100 runs. Table 2 provides the average estimates and their SEs across runs (more detailed simulation results can be found in Section J in S1 Text). Across scenarios and runs, estimates are close to the true values and have small SEs. For the scenario with partial mediation (*Baseline*), estimates indeed reveal both the direct and indirect effect; for the scenarios with no mediation (*i*–*iii*), the estimated indirect effect is indeed very close to zero; for the scenario with full mediation (*iv*), the estimated direct effect is very close to zero, as expected. We conclude that MA-GREML consistently estimates the effect of *M* on *Y*, the direct effect, and the indirect effect, irrespective of whether we have no mediation, partial mediation, or full mediation.

**Table 2:** Average estimates and corresponding standard errors of estimated parameters in the mediation model (100 simulation runs for each scenario). $\hat{b}$ is the estimator of *b* based on Eq 8 (i.e., the effect of mediator *M* on outcome *Y*); $\hat{\text{direct}\;\text{effect}}$ is an estimator of *c*^2^ based on Eq 10 (i.e., the genetic variance of *Y* that is not mediated by *M*); $\hat{\text{indirect}\;\text{effect}}$ is an estimator of (*a*^2^ + *g*^2^)*b*^2^ based on Eq 9 (i.e., the genetic variance of *Y* that is mediated by *M*); average standard errors are reported between parentheses.

Scenario	True parameter values	$\hat{b}$	$\hat{\text{direct}\;\text{effect}}$	$\hat{\text{indirect}\;\text{effect}}$
*Baseline*	*b* = 1, *c*^2^ = 1, (*a*^2^ + *g*^2^) = 2	0.999	1.000	1.996
		(0.013)	(0.040)	(0.061)
(*i*)	*b* = 1, *c*^2^ = 1, (*a*^2^ + *g*^2^) = 0	0.999	0.999	0.003
		(0.011)	(0.026)	(0.010)
(*ii*)	*b* = 0, *c*^2^ = 1, (*a*^2^ + *g*^2^) = 2	-0.001	1.000	0.000
		(0.013)	(0.040)	(0.000)
(*iii*)	*b* = 0, *c*^2^ = 1, (*a*^2^ + *g*^2^) = 0	-0.001	0.999	0.000
		(0.011)	(0.026)	(0.000)
(*iv*)	*b* = 1, *c*^2^ = 0, (*a*^2^ + *g*^2^) = 2	0.997	0.004	1.990
		(0.012)	(0.010)	(0.063)

**Fig 2:**
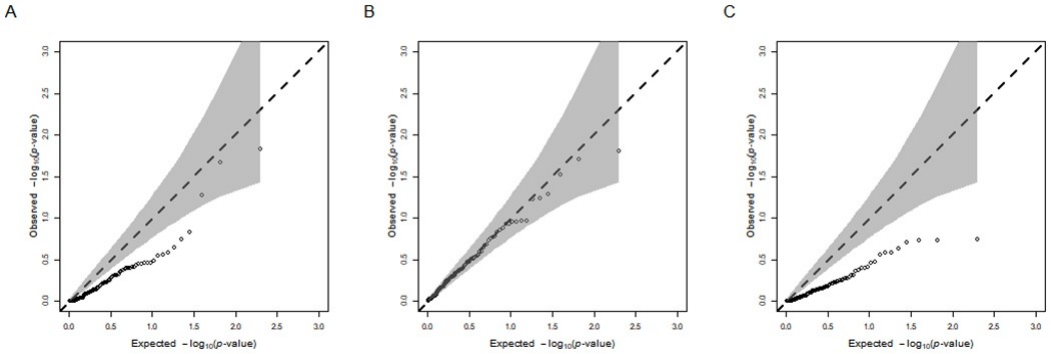
QQ-plots visualizing the distribution of *p*-values resulting from the likelihood ratio tests (LRTs) for the presence of the indirect effect (*a*^2^ + *g*^2^)*b*^2^ in different scenarios. Panel (a): Mediator *M* does not have a genetic component, but has an effect on outcome *Y* ((*a*^2^ + *g*^2^) = 0, *b* ≠ 0); Panel (b): Mediator *M* has a genetic component, but does not have an effect on outcome *Y* ((*a*^2^ + *g*^2^) > 0, *b* = 0); Panel (c): Mediator *M* does not have a genetic component, nor an effect on outcome *Y* ((*a*^2^ + *g*^2^) = 0, *b* = 0).

Regarding the behavior of our LRT for the indirect effect, in Scenario (*i*) the optimum of the likelihood of the model under the null hypothesis (i.e., under the constraint that (*a*^2^ + *g*^2^) *b*^2^ = 0) is typically found in $P_{a}$ (i.e., where *a*^2^ + *g*^2^ = 0 and where *b* can differ from zero) and, therefore, the LRT tends to have two degree of freedom. Fig 2A shows the observed distribution of *p*-values resulting from the LRTs compared to the expected distribution of *p*-values. We find that the observations lie somewhat below the 45 degree line. Thus, the LRT is conservative in case *M* has no genetic variance at all, implying a reduced false-positive rate in that case. By the same token, this finding implies that the LRT is underpowered in case *M* has a very low but non-zero $h_{\text{SNPs}}^{2}$. This drawback, however, has little practical relevance, as users would typically only consider potential mediators that have an appreciable $h_{\text{SNPs}}^{2}$.

In Scenario (*ii*), the optimum of the likelihood of the model under the null hypothesis is mostly found in $P_{b}$ (i.e., where *b* = 0 and where *a*^2^ + *g*^2^ can exceed zero) and, therefore, the LRT tends to have one degree of freedom. Fig 2B plots the observed distribution of *p*-values resulting from the LRTs compared to the expected distribution of *p*-values. With the observations being close to the diagonal, we find that the empirically observed *p*-values closely follow their theoretically expected distribution in case *b* = 0 and (*a*^2^ + *g*^2^)>0. Thus, also in this setting, we do not suffer from an inflated false-positive rate. Moreover, here we find no evidence of any loss in statistical power (e.g., where *b* is small but nonzero). Within the set of scenarios where the null hypothesis of no indirect effect holds true (i.e., Scenarios *i*–*iii*), we judge this scenario to be the most realistic one because in practice mediators will have a non-negligible $h_{\text{SNPs}}^{2}$, irrespective of whether they affect *Y*.

Finally, for Scenario (*iii*) the observed distribution of *p*-values resulting from the LRTs compared to the expected distribution of *p*-values are shown in Fig 2C. In this setting, it is not possible to *a priori* determine whether the optimum of the likelihood of the model under the null hypothesis will be in either $P_{a}$ or $P_{b}$. This absence of a prior expectation for the degrees of freedom poses no problem; MA-GREML simply sets the degrees of freedom based on where the optimum is found under the restrictions imposed by the null hypothesis (i.e., two degrees of freedom if the optimum under the null hypothesis is found in $P_{a}$ and one if found in $P_{b}$). The observed *p*-values, like in Scenario (*i*), are somewhat conservative. We conclude that also in this scenario the procedure for testing the significance of the indirect effect sufficiently controls for Type I errors. Again, we note that in practice a setting in which the mediator is not heritable is not realistic.

## Applications

### Ethics statement

For our empirical illustrations, we employ data from the US Health and Retirement Study (HRS). The HRS is a longitudinal panel study that surveys a representative sample of approximately 20,000 individuals aged 51 years and older (and their spouses) in the United States of America [27]. Collection and production of HRS data comply with the requirements of the University of Michigan’s Institutional Review Board (HUM0006112). The specific research project in which the present study is embedded has been approved locally by the Institutional Review Board of the Erasmus Research Institute of Management (IRB-E 2014–04).

### Empirical results

To construct the genomic-relatedness matrix (GRM) we need for the GREML analyses, we use the 2012 release of genetic data. These genetic data were obtained from the DNA samples collected from HRS participants in the years between 2006 and 2008. Participants signed consent forms stating that the genetic data could be used for non-profit research use only. Genotyping was carried out using the Illumina Human Omni-2.5 Quad BeadChip. After imposing the quality control filters recommended by the genotyping center, removal of non-autosomal SNPs and SNPs with minor allele frequency (MAF) < 0.05, genotyping call rate < 95% and Hardy–Weinberg *p* < 10^−6^, and selection of unrelated individuals from European ancestry with <5% SNP missingness, we construct the GRM for a sample of 8,652 individuals using 1,188,298 SNPs.

Phenotypic data from the biennial waves of data collection in the HRS are harmonized by the RAND cooperation. We exploit information about educational attainment (years) and four health outcomes in later life—Body Mass Index (BMI; kg/m^2^), Cognition (index score ranging from 0–35), Mental health (index score ranging from 0–8), and Self-reported health (index score ranging from 1–5)—as available in the RAND HRS Longitudinal File 2018 (V1). By analyzing a variable that is realized in early life as mediator and health measures in later life as outcomes, we make sure that the mediating variable precedes the outcomes. We selected phenotypic data from the wave of data collection with the (combined) lowest number of missing values for these four outcomes (Wave 8). In addition, we analyze to what extent genetic effects run through an earlier realization of these four variables. That is, we analyze whether the Wave 7 measures mediate the additive genetic effect on the Wave 8 outcomes.

There are 5,305–8,565 individuals with information on the four later-life health outcomes. For the mediators, the sample size ranges between 5,747–8,638. In our analyses, we control for sex (Female/Male) and birth year. MGREML also adjusts for the first 20 principal components of the genomic-relatedness matrix to control for subtle forms of population stratification [37, 38]. Table 3 provides descriptive statistics of the HRS analysis sample. Due to the exclusion of individuals with a genetic relatedness larger than 0.025 in the analysis sample, the actual analysis samples comprise slightly fewer individuals (see Table 4).

**Table 3:** Descriptive statistics of the analysis sample from the Health and Retirement Study. Std. dev. = Standard deviation; Min. = Minimum; Max. = Maximum.

Variables	*N*	Mean	Std. dev.	Min.	Max.
*Outcomes*					
Body Mass Index (kg/m^2^)	8,485	27.703	5.596	10.6	82.7
Cognition (index)	5,305	22.754	4.501	0.0	35.0
Mental health (index)	8,521	1.257	1.824	0.0	8.0
Self-reported health (index)	8,565	2.655	1.058	1.0	5.0
*Mediators*					
Educational attainment (years)	8,638	13.154	2.541	0.0	17.0
Body Mass Index (kg/m^2^)—Lagged	8,340	27.369	5.355	13.7	60.1
Cognition (index)—Lagged	5,747	23.675	4.066	3.0	35.0
Mental health (index)—Lagged	8,195	1.168	1.745	0.0	8.0
Self-reported health (index)—Lagged	8,424	2.609	1.058	1.0	5.0
*Covariates*					
Sex (1 = Female; 0 = Male)	8,652	0.584	0.493	0	1
Birth year	8,652	1937.831	10.424	1905	1974

**Table 4:** MA-GREML results for the empirical analyses using data from the Health and Retirement Study. LRT = Likelihood-Ratio Test for significance of indirect effect (*a*^2^ + *g*^2^)*b*^2^ (i.e., the proportion of $h_{\text{SNPs}}^{2}$ of outcome *Y* running through mediator *M*); Standard errors in parentheses.

Outcome (*Y*)	Mediator (*M*)	*N*	$h_{\text{SNPs}}^{2}$ (*Y*)	$h_{\text{SNPs}}^{2}$ (*M*)	Effect *M* on *Y* (*b*)	Prop. $h_{\text{SNPs}}^{2}\left(Y\right)$ through *M*	LRT *p*-value
Body Mass Index	Educational attainment	8,213	0.187 (0.047)	0.263 (0.046)	-0.222 (0.100)	1.4%	0.025
Cognition	Educational attainment	5,169	0.242 (0.074)	0.298 (0.073)	0.323 (0.118)	27.6%	0.011
Mental health	Educational attainment	8,250	0.192 (0.046)	0.265 (0.046)	-0.044 (0.033)	7.3%	0.182
Self-reported health	Educational attainment	8,291	0.106 (0.045)	0.272 (0.046)	-0.046 (0.019)	30.6%	0.010
Body Mass Index	Body Mass Index—Lagged	8,005	0.163 (0.048)	0.189 (0.048)	0.942 (0.019)	99.9%	3.615 × 10^−4^
Cognition	Cognition—Lagged	4,334	0.251 (0.088)	0.256 (0.088)	0.542 (0.073)	71.3%	2.901 × 10^−3^
Mental health	Mental health—Lagged	7,915	0.181 (0.048)	0.086 (0.047)	0.500 (0.033)	54.9%	1.563 × 10^−3^
Self-reported health	Self-reported health—Lagged	8,129	0.126 (0.047)	0.192 (0.047)	0.665 (0.030)	96.8%	7.785 × 10^−5^

The main results of the empirical analyses are presented in Table 4. The later-life health outcomes are all heritable. The SNP-based heritability of BMI is 18.7%, and that of cognition is 24.2%. It equals 19.2% for mental health and 10.6% for self-reported health. The heritability estimates of the mediator (educational attainment) ranges from 26.3% to 29.8% across the slightly different analysis samples. The estimated effects *b* of *M* on *Y* show that every additional year of educational attainment leads to a decrease in BMI of 0.222 kg/m^2^ and an increase in the cognition index score of 0.323. For mental health and self-reported health, the estimates are −0.044 and −0.046, respectively. We find that the effect of the additive genetic factor of BMI on BMI runs through educational attainment for just 1.4%, but this indirect effect is statistically significant (*p* = 0.025). For mental health, the indirect effect appears to be statistically insignificant (*p* = 0.182). The proportion mediated for cognition is 27.6% (*p* = 0.011). This percentage is roughly similar for self-reported health, 30.6%, and for this outcome the indirect effect is also significant (*p* = 0.010).

For the analyses with the lagged variables as mediators, the results are shown in the bottom of Table 4. For BMI, we observe full mediation: the proportion mediated is 99.9%, and the indirect effect is statistically significant (*p* = 3.615 × 10^−4^). For self-reported health, we observe a similarly high proportion mediated, i.e., 96.8% (*p* = 7.785 × 10^−5^). These estimates suggest that the additive genetic factor of these two outcomes is relatively time-invariant between consecutive data collection waves in the analysis sample. For cognition and mental health the proportions mediated are lower, 71.3% (*p* = 2.901 × 10^−3^) and 54.9% (*p* = 1.563 × 10^−3^), respectively, suggesting relatively strong impact of the environment on the development of these traits over the life-course.

## Discussion

We designed a framework to analyze to what extent a particular factor mediates the relationship between the additive genetic factor of a trait and the trait itself. By doing so, we particularly improve on the current practice of using polygenic scores for such analyses, because GREML enables capturing the full SNP-based heritability of a trait rather than just the ‘explained SNP-based heritability’ [15]. The statistical procedure has been implemented in the ready-to-use command-line tool MGREML [19]. The GitHub page (https://github.com/devlaming/mgreml) accompanying this tool comes with a full tutorial on its usage.

Usage of MGREML for mediation analysis requires careful assessment of whether the assumptions of the model hold for the specific empirical application, in particular that the causal direction between mediator *M* and outcome *Y* is correctly specified. Moreover, SNP-based heritability estimates may capture both direct genetic effects and gene-environment correlations such as genetic nurture effects (i.e., the effect of parental genotype on the child’s outcomes), depending on the outcome analyzed [15, 39]. In Section H in S1 Text we introduce a structural equation model including genetic nurture effects and we derive that in situations where parental factors (e.g., parental genes or parental educational attainment) affect offspring outcomes estimates of mediating effects are typically affected. As genetic nurture can lead to both over and underestimation of the effect of mediator *M* on outcome *Y*, the impact of such effects on the MA-GREML estimates depends on the specific empirical application.

Nevertheless, the assessment of mediation is of prime importance in the social sciences [1], because it allows for an improved understanding of the mechanisms underlying the relationship between a predictor and an outcome. As a result, more nuanced questions extending beyond merely determining whether an outcome occurs can be analyzed and answered. However, the results of mediation analyses are also informative for genetic epidemiology. The proxy-phenotype approach has been developed to discover genetic variants in a GWAS for being associated with a trait by investigation of a closely related but easier to measure trait [26]. While a strong genetic correlation between the two traits often serves as a motivation for this experimental design [36], backing up such a study with an analysis showing that genetic effects on the easier to measure trait in fact largely run through the more difficult to measure trait would particularly build confidence in the eventual proxy-GWAS findings.

Moreover, Mendelian randomization (MR) models are widely used for causal inferences by exploiting the notion that the inheritance of alleles is random conditional of the genotypes of the parents [24]. In MR models, the exclusion assumption needs to hold meaning that the main genetic factor which is used as instrument is only allowed to impact the outcome variable through the mediator. Whether this assumption holds in empirical applications is often debatable and not empirically verifiable [24, 25], especially when a PGS based on a genome-wide scan of SNPs is being used as instrument. However, our mediation model may provide suggestive evidence in favor of the exclusion restriction to hold in case full mediation is found.

Even in case a biology-informed decision is taken regarding the subset of genetic variants to use in MR applications, the GREML-based mediation model can be used for such an informative analysis by constructing a GRM using these genetic variants only rather than a genome-wide scan of SNPs. For example, the GRM can be constructed using SNPs from specific chromosomes, SNPs in genes expressed in particular cell types (functional categories), or SNPs with an allele frequency in a particular range, etc. [40–42]. Moreover, SNPs included in the GRM can be weighted to reflect a specific heritability model [18, 43]. As illustration, Section K in S1 Text contains analysis results for the four later-life outcomes from the empirical illustration with educational attainment as mediator for which we partitioned the GRM by ten functional categories. Interestingly, we find that the evidence for mediation differs across functional categories.

The empirical results from the Health and Retirement Study presented in the main text, however, suggest that for two reasons MR analysis based on a genome-wide scan of SNPs (or a PGS as summary of that) cannot be used to analyze the causal impact of educational attainment on BMI, cognition, mental health, or self-reported health in later life. While for BMI, cognition and self-reported health we do find a significant indirect effect, the proportion mediated is (far) below 100% suggesting that there are other channels than educational attainment through which the additive genetic factor for these two outcomes affects the outcomes. For mental health the indirect effect is statistically insignificant, both invalidating the exclusion restriction and the so-called relevance assumption that the instrument has explanatory power for the mediator [24]. In the empirical analyses with lagged versions of the outcome variable as mediator, we find that the additive genetic factor underlying the outcome can be time-variant for some traits. These results underscore that the conventional assumption in MR analyses that the effects of the genetic variants used as instruments on the exposure (i.e., the mediator in the MA-GREML framework) do not vary over the life-course is often questionable [44].

Thus, beyond the assessment of the relative size of indirect effects, these results show that results obtained using MA-GREML are of importance for validating assumptions of other methodologies. Therefore, we believe that the mediation analysis approach as developed in this study and implemented in the freely available MGREML tool will prove to be an important framework not only for the social sciences but also for genetic epidemiology.

## Supporting information

S1 Text Derivations of the model, a comparison with Genomic SEM, a study of the model under genetic nurture, simulation set-up, simulation results. (PDF)

S1 Data Full simulation output. (XLSX)
