# Supplementary material for: The identification of mediating effects using genome-based restricted maximum likelihood estimation
Source: PLoS Genet. 2023 Feb 21;19(2):e1010638. doi: 10.1371/journal.pgen.1010638 (PMC9983879; doi:10.1371/journal.pgen.1010638)
Supplement: S1 Text — (PDF) [file pgen.1010638.s001.pdf]

Supplementary Information "The identification of mediating effects using Genome-based Restricted Maximum Likelihood estimation"

Cornelius A. Rietveld[1,2], Ronald de Vlaming[3], and Eric A. W. Slob[1,2,4,*]

1 Department of Applied Economics, Erasmus School of Economics, Erasmus University Rotterdam, Rotterdam, The Netherlands

2 Erasmus University Rotterdam Institute for Behavior and Biology, Erasmus School of Economics, Erasmus University Rotterdam, Rotterdam, The Netherlands

3 Department of Economics, School of Business and Economics, Vrije Universiteit Amsterdam, Amsterdam, The Netherlands

4 Medical Research Council Biostatistics Unit, School of Clinical Medicine, University of Cambridge, Cambridge, United Kingdom

* eric.slob@mrc-bsu.cam.ac.uk

Section A The model and the main quantities of interest

Our main structural model is shown in Fig A. This model underpins our method that we refer to as MA-GREML: Mediation Analysis using Genome-based Restricted Maximum Likelihood (GREML). In this model, G and G^* are genetic components, M is the mediating factor, E and E^* are environmental components, and Y is the outcome. Without loss of generality, all latent variables (i.e., G, G^*, E, E^*) are assumed to have mean zero and both E and E^* are assumed to have unit variance. The covariance of E (E^*) between two individuals is equal to zero (i.e., there is no correlation in environmental factors across individuals). The covariance structure of both G and G^* is shaped directly by the so-called genomic-relatedness matrix (GRM). That is, the

variance of G (resp. G^*) for a given individual equals the corresponding diagonal element from the GRM and the covariance of G (G^*) between two individuals is equal to the corresponding off-diagonal element of the GRM. Moreover, these four latent variables are assumed to be uncorrelated. In addition, we stress that both G and G^* constitute 'additive genetic factors': they are aggregates across single-nucleotide polymorphisms (SNPs), and not about individual SNPs. More technically, G and G^* can be conceptualised as linear combinations of all available SNPs. If G and G^* were to be observed at the individual level, these would constitute so-called breeding values.

The model comprises six path coefficients. Our study revolves around two core quantities that can be defined as functions of these path coefficients. First, we go over the definition of these core quantities. Second, we provide intuition about what these quantities reflect. The definitions are as follows:

1. The mediated genetic variance of Y: $\left(a^2 + g^2\right) b^2$;
2. The non-mediated genetic variance of Y: c^2.

From the perspective of a classical regression-based mediation analysis, (i) the mediated genetic variance of Y can be conceptualised as the *indirect effect* and (ii) the non-mediated genetic variance of Y can be thought of as the *direct effect*. Both effects are about the contribution of additive, random effects of SNPs to variation in Y. From the perspective of the literature on classical mediation analysis, these quantities could also be referred to as squared direct/indirect effects. However, for brevity we just refer to these quantities as the direct and indirect effect, both here and in the main text.

The indirect effect is zero if $a = g = 0$ or $b = 0$ (or both). In this case, the genetic variance of Y is not mediated by M. The direct effect is zero if $c = 0$. In that case, the genetic variance of Y is fully mediated by M. If both the indirect and direct effect are zero, outcome Y has no genetic variance. In that case, the SNP-based heritability (h^2_{SNPs}) of Y is zero. Finally, in case $b \neq 0$, $c \neq 0$, and $a \neq 0$ and/or $g \neq 0$, the genetic variance of Y is partially mediated by M.

A conceptual complication arises here because we analyze contributions to variances rather than contributions to expectations. More specifically, if the genetic component of Y can be written as $\delta + \gamma$, where δ denotes the direct component and γ the indirect component, then the genetic variance of Y equals $\text{Var}(\delta) + \text{Var}(\gamma) + 2\text{Cov}(\delta, \gamma)$, where

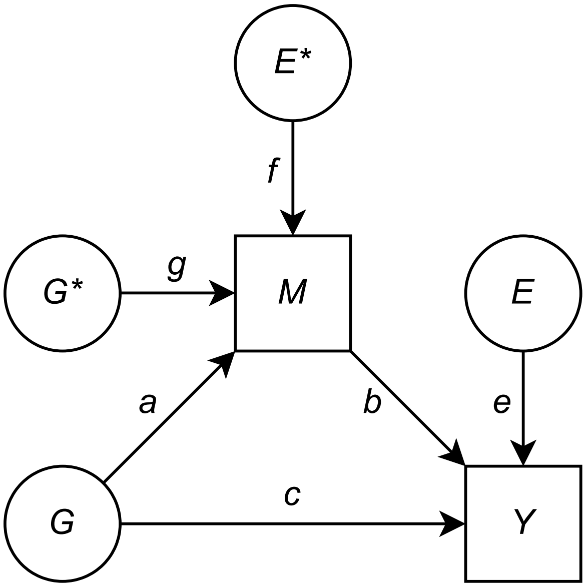

Fig A. Structural equation model (SEM) for mediation analysis using GREML (MA-GREML), to quantify to which extent the genetic component of trait Y affects trait Y through trait M. G and G^* are latent genetic factors, E and E^* are latent environmental factors, M is the observed mediator, Y is the observed outcome, $(a^2+g^2)b^2$ is the genetic variance of Y that is mediated by M (*indirect effect*), and c^2 is the genetic variance of Y that is not mediated by M (*direct effect*); *Full mediation*: direct effect $= 0$ and indirect effect > 0; *Partial mediation*: direct and indirect effect both > 0; *No mediation*: direct effect > 0 and indirect effect $= 0$.

$\mathrm{Var}(\delta) = c^2$, $\mathrm{Var}(\gamma) = \left(a^2+g^2\right)b^2$, and $\mathrm{Cov}(\delta,\gamma)$ is the covariance between the direct and indirect component. Clearly, the model in Fig A allows for a non-zero covariance between δ and γ, as G affects Y directly as well as indirectly via M whenever $abc \neq 0$. Thus, although $\left(a^2+g^2\right)b^2$ reflects the indirect effect and c^2 the direct effect, the total genetic variance of Y will typically differ from the sum of these two parts. This property of our model is presented more formally in Section B.

Section B Structural equations and implied variances and covariances

The model in Fig A simply assumes that M is a linear combination of G, G^*, and E^*, and in turn that Y is a linear combination of M, G, and E. More specifically, the

following two structural equations hold for M and Y:

$$M = Ga + G^{*}g + E^{*}f; \tag{1}$$

$$Y = Mb + Gc + Ee. \tag{2}$$

Now, by substituting M in Eq. 2 by the right-hand side of Eq. 1, we can rewrite the equation for Y as follows:

$$Y = \left(Ga + G^{*}g + E^{*}f\right)b + Gc + Ee \tag{3}$$

$$= Gab + G^{*}gb + E^{*}fb + Gc + Ee \tag{4}$$

$$= G\left(ab + c\right) + G^{*}gb + E^{*}fb + Ee. \tag{5}$$

Thus, we now have equations for both M and Y describing them as linear combinations of only the latent components G, G^{*}, E, and E^{*}. Recall that these components are assumed to be uncorrelated, have mean zero, and unit variance. Moreover, we assume fixed parameters (i.e., non-random path coefficients). Under these assumptions (and using the identities $\mathbb{E}\left[A + B\right] = \mathbb{E}\left[A\right] + \mathbb{E}\left[B\right]$ and $\mathbb{E}\left[Aw\right] = \mathbb{E}\left[A\right]w$ for random variables A and B and scalar w) we have that

$$\mathbb{E}\left[M\right] = \mathbb{E}\left[G\right]a + \mathbb{E}\left[G^{*}\right]g + \mathbb{E}\left[E^{*}\right]f = 0a + 0g + 0f \equiv 0, \text{ and} \tag{6}$$

$$\mathbb{E}\left[Y\right] = \mathbb{E}\left[G\right]\left(ab + c\right) + \mathbb{E}\left[G^{*}\right]gb + \mathbb{E}\left[E^{*}\right]fb + \mathbb{E}\left[E\right]e \tag{7}$$

$$= 0\left(ab + c\right) + 0gb + 0fb + 0e \equiv 0. \tag{8}$$

That is, mediator M and outcome Y have mean zero. Now, recalling a standard result from probability theory that for a random variable A with $\mathbb{E}\left[A\right] = 0$ we have that $\mathrm{Var}\left(A\right) = \mathbb{E}\left[A^{2}\right]$, we can write the variance of M as follows:

$$\mathrm{Var}\left(M\right) = \mathbb{E}\left[\left(Ga + G^{*}g + E^{*}f\right)^{2}\right] \tag{9}$$

$$= \mathbb{E}\left[G^{2}a^{2} + GG^{*}2ag + GE^{*}2af + \left(G^{*}\right)^{2}g^{2} + G^{*}E^{*}2gf + \left(E^{*}\right)^{2}f^{2}\right] \tag{10}$$

$$= \mathbb{E}\left[G^{2}\right]a^{2} + \mathbb{E}\left[GG^{*}\right]2ag + \mathbb{E}\left[GE^{*}\right]2af + \mathbb{E}\left[\left(G^{*}\right)^{2}\right]g^{2} + \mathbb{E}\left[G^{*}E^{*}\right]2gf + \mathbb{E}\left[\left(E^{*}\right)^{2}\right]f^{2}. \tag{11}$$

Recalling another result from probability theory which states $\operatorname{Cov}(A, B) = \mathbb{E}[AB]$ for random variables A and B in case $\mathbb{E}[A] = \mathbb{E}[B] = 0$, we can rewrite the variance of M as follows:

$$\begin{aligned}
\operatorname{Var}(M) = {} & \operatorname{Var}(G)\, a^2 + \operatorname{Cov}(G, G^*)\, 2ag + \operatorname{Cov}(G, E^*)\, 2af + \operatorname{Var}(G^*)\, g^2 \\
& + \operatorname{Cov}(G^*, E^*)\, 2gf + \operatorname{Var}(E^*)\, f^2 && (12) \\
= {} & \operatorname{Var}(G)\, a^2 + \operatorname{Var}(G^*)\, g^2 + \operatorname{Var}(E^*)\, f^2 && (13) \\
= {} & 1a^2 + 1g^2 + 1f^2 && (14) \\
= {} & a^2 + g^2 + f^2. && (15)
\end{aligned}$$

Notice that all the covariance terms between latent components on the right-hand side have disappeared because these latent components are assumed to be uncorrelated. This disappearance of covariance terms also applies when deriving the implied variance of Y as well as the covariance between M and Y. In further derivations, to avoid undue repetition, we skip the intermediate step that makes covariances between latent components explicit. Also observe that $\operatorname{Var}(G) = \operatorname{Var}(G^*) = 1$, as the genomic relatedness of an individual with itself is on average equal to one.

For the genetic variance and environmental variance of M we have that

$$\operatorname{Var}_G(M) = a^2 + g^2, \text{ and} \quad (16)$$

$$\operatorname{Var}_E(M) = f^2. \quad (17)$$

Analogously, for Y we have

$$\operatorname{Var}(Y) = \mathbb{E}\left[(G(ab+c) + G^* gb + E^* fb + Ee)^2\right] \tag{18}$$

$$= \operatorname{Var}(G)(ab+c)^2 + \operatorname{Var}(G^*) g^2 b^2 + \operatorname{Var}(E^*) f^2 b^2 + \operatorname{Var}(E) e^2 \tag{19}$$

$$= 1(ab+c)^2 + 1g^2b^2 + 1f^2b^2 + 1e^2 \tag{20}$$

$$= (ab+c)^2 + g^2b^2 + f^2b^2 + e^2, \tag{21}$$

$$\operatorname{Var}_G(Y) = (ab+c)^2 + g^2b^2 \tag{22}$$

$$= a^2b^2 + c^2 + 2abc + g^2b^2 \tag{23}$$

$$= \left(a^2 + g^2\right) b^2 + c^2 + 2abc, \text{ and} \tag{24}$$

$$\operatorname{Var}_E(Y) = f^2b^2 + e^2. \tag{25}$$

Here, it can be observed that the genetic variance of Y indeed comprises the direct effect (i.e., c^2), the indirect effect (i.e., $\left(a^2 + g^2\right) b^2$), as well as the previously alluded to covariance term, which equals $2abc$. For the covariance of M and Y, we have that

$$\operatorname{Cov}(M, Y) = \mathbb{E}[MY] \tag{26}$$

$$= \mathbb{E}\left[(Ga + G^* g + E^* f)(G(ab+c) + G^* gb + E^* fb + Ee)\right] \tag{27}$$

$$= \mathbb{E}\left[G^2\right] a(ab+c) + \mathbb{E}\left[(G^*)^2\right] g^2 b + \mathbb{E}\left[(E^*)^2\right] f^2 b \tag{28}$$

$$= \operatorname{Var}(G) a(ab+c) + \operatorname{Var}(G^*) g^2 b + \operatorname{Var}(E^*) f^2 b \tag{29}$$

$$= 1a(ab+c) + 1g^2b + 1f^2b \tag{30}$$

$$= a(ab+c) + g^2b + f^2b \tag{31}$$

$$= a^2b + ac + g^2b + f^2b \tag{32}$$

$$= \left(a^2 + g^2 + f^2\right) b + ac. \tag{33}$$

Moreover, the genetic covariance and environmental covariance between M and Y are as follows:

$$\operatorname{Cov}_G(M, Y) = \left(a^2 + g^2\right) b + ac, \text{ and} \tag{34}$$

$$\operatorname{Cov}_G(E, Y) = f^2 b. \tag{35}$$

These expressions immediately reveal that it is not possible to use the ratio of the total covariance and the total variance of M to obtain b. After all, for that ratio we have:

$$\frac{\mathrm{Cov}\left(M,Y\right)}{\mathrm{Var}\left(M\right)} = \frac{\left(a^2+g^2+f^2\right)b+ac}{a^2+g^2+f^2} \tag{36}$$

$$= b + \frac{ac}{a^2+g^2+f^2} \neq b. \tag{37}$$

This formulation reveals that G effectively serves as a confounder of the association between M and Y when considering their total covariance. Thus, a standard approach such as ordinary least squares (OLS) to estimate the regression coefficient of M with respect to Y will fail to consistently estimate b. This observation brings us to the core of our identification strategy: We use the environmental variance and covariance to obtain an estimator of b. More specifically, we have that:

$$\frac{\mathrm{Cov}_E\left(M,Y\right)}{\mathrm{Var}_E\left(M\right)} = \frac{f^2 b}{f^2} \equiv b. \tag{38}$$

The crucial assumption underpinning this strategy is that our fixed-effect covariates (i.e., factors that we control for in the estimation procedure) include all relevant confounders. Indeed, inter-generational transfers and cultural transmissions pose a big challenge to mediation models such as the one presented here. This limitation will be further discussed in Section H.

Section C From variance components to path coefficients

As in the main text, let σ^G_{MM} denote the genetic variance of M, σ^G_{MY} the genetic covariance of M and Y, and let σ^E_{MM} and σ^E_{MY} be defined analogously for the environmental (co)variance. Finally, let σ^G_{YY} (resp. σ^E_{YY}) denote the genetic (environmental) variance of Y. Also, observe that these genetic variances and covariances can be readily estimated using the software tools GCTA and MGREML. The expressions for genetic and environmental (co)variances found in Section B can be

summarised as follows:

$$\sigma^G_{MM} = a^2 + g^2, \tag{39}$$

$$\sigma^G_{YY} = \left(a^2 + g^2\right) b^2 + c^2 + 2abc, \tag{40}$$

$$\sigma^G_{MY} = \left(a^2 + g^2\right) b + ac, \tag{41}$$

$$\sigma^E_{MM} = f^2, \tag{42}$$

$$\sigma^E_{YY} = f^2 b^2 + e^2, \tag{43}$$

$$\sigma^E_{MY} = f^2 b. \tag{44}$$

Section C.1 Identification

For MA-GREML, the path coefficient are not of prime interest. Rather, we are interested in the direct effect and the indirect effect. Thus, we only need to find expressions for the path coefficients (or transformations thereof) that are needed to identify those two effects. In fact, in our model, only path coefficient b is fully identified, provided $f \neq 0$ (i.e., there is environmental variance in M—a requirement we assume to hold in the derivations). Path coefficients c, f, and e are always identified, except for the sign (e.g., a model which gives $\widehat{e}$ as estimate for e has identical fit to a model returning $-\widehat{e}$ as estimate for e). Put differently, in our model b, c^2, f^2, and e^2 are uniquely identified. Interestingly, in case $c = 0$ (i.e., the direct effect is zero), we can identify $a^2 + g^2$, but we cannot identify a^2 and g^2 separately. However, this seeming lack of identification when $c = 0$ has no bearing on the identification of the direct effect and the indirect effect.

Section C.2 Mapping of variance components to path coefficients

Here, using Eq 39–44, we map the variance components (VCs) to a^2, b, c^2, e^2, f^2, and g^2. The estimate for f^2 is most straightforward to derive, and equals the environmental component of mediator M:

$$f^2 = \sigma^E_{MM}. \tag{45}$$

Using this equivalence, we obtain:

$$b = \frac{\sigma_{MY}^E}{f^2} = \frac{\sigma_{MY}^E}{\sigma_{MM}^E}. \tag{46}$$

By combining the expression for the environmental variance of Y with Eq 45, and Eq 46, we obtain:

$$e^2 = \sigma_{YY}^E - f^2 b^2 = \sigma_{YY}^E - \frac{(\sigma_{MY}^E)^2}{\sigma_{MM}^E}. \tag{47}$$

To solve for c^2, we first observe from substituting the expression for σ_{MY}^G into the expression for σ_{YY}^G that:

$$c^2 = \sigma_{YY}^G - \sigma_{MY}^G b - abc. \tag{48}$$

An expression for abc can be obtained by substituting the expression for σ_{MM}^G into the expression for σ_{MY}^G:

$$ac = \sigma_{MY}^G - \sigma_{MM}^G b, \tag{49}$$

and by multiplying both sides of the resulting expression by b:

$$abc = \sigma_{MY}^G b - \sigma_{MM}^G b^2. \tag{50}$$

Insertion of Eq 48 into Eq 50 yields:

$$c^2 = \sigma_{YY}^G - \sigma_{MY}^G b - \left(\sigma_{MY}^G b - \sigma_{MM}^G b^2\right) = \sigma_{YY}^G + \sigma_{MM}^G b^2 - 2\sigma_{MY}^G b. \tag{51}$$

With our expression for b from Eq 46, we obtain:

$$c^2 = \sigma_{YY}^G + \sigma_{MM}^G \left(\frac{\sigma_{MY}^E}{\sigma_{MM}^E}\right)^2 - 2\sigma_{MY}^G \frac{\sigma_{MY}^E}{\sigma_{MM}^E}. \tag{52}$$

Using the expressions for c^2 and ac, and given $c^2 > 0$ we can derive that:

$$a^2 = a^2 \frac{c^2}{c^2} = \frac{(ac)^2}{c^2} = \frac{\left(\sigma_{MY}^G - \sigma_{MM}^G \frac{\sigma_{MY}^E}{\sigma_{MM}^E}\right)^2}{\sigma_{YY}^G + \sigma_{MM}^G \left(\frac{\sigma_{MY}^E}{\sigma_{MM}^E}\right)^2 - 2\sigma_{MY}^G \frac{\sigma_{MY}^E}{\sigma_{MM}^E}}. \tag{53}$$

Finally, we obtain from the expression for σ^G_{MM}:

$$g^2 = \sigma^G_{MM} - a^2 = \sigma^G_{MM} - \frac{\left(\sigma^G_{MY} - \sigma^G_{MM}\frac{\sigma^E_{MY}}{\sigma^E_{MM}}\right)^2}{\sigma^G_{YY} + \sigma^G_{MM}\left(\frac{\sigma^E_{MY}}{\sigma^E_{MM}}\right)^2 - 2\sigma^G_{MY}\frac{\sigma^E_{MY}}{\sigma^E_{MM}}}. \tag{54}$$

Together, these expressions facilitate a mapping from the VCs being estimated using bivariate GREML to the parameters of the structural model in Fig A.

Section C.3 The direct effect and indirect effect

We now use the mapping of VCs to path coefficients to write the direct effect and the indirect effect as functions of the variance components. The direct effect of the additive genetic component of Y that does not run through M is given by c^2. We have derived an expression for this in Eq 52:

$$c^2 = \sigma^G_{YY} + \sigma^G_{MM}\left(\frac{\sigma^E_{MY}}{\sigma^E_{MM}}\right)^2 - 2\sigma^G_{MY}\frac{\sigma^E_{MY}}{\sigma^E_{MM}}. \tag{55}$$

The effect of the additive genetic component of Y on Y through M is given by $(a^2 + g^2)b^2$. Using the expressions for a^2, b, and g^2 from Eq 46, Eq 53, and Eq 54, we can rewrite this indirect effect as:

$$(a^2 + g^2)b^2 = \sigma^G_{MM}\left(\frac{\sigma^E_{MY}}{\sigma^E_{MM}}\right)^2. \tag{56}$$

Observe that for identification of the direct and indirect effect, we only require $f \neq 0$ (i.e., there is environmental variance in M). In case $c = 0$ (i.e., full mediation), all path coefficients are still identified, except for a^2 and g^2. However, even in this case, $a^2 + g^2$ is still identified. Therefore, even if there is full mediation, b, the direct effect, and the indirect effect can all still be quantified.

Section C.4 Variances of the estimated parameters of interest

A bivariate GREML model that properly controls for confounding variables enables the estimation of VCs that can be transformed to estimates of the parameters in the underlying structural model. Here, we use a delta method to obtain the approximate variance (standard error; SE) of the estimators of prime interest: The genetic variance

of outcome Y and mediator M, the indirect effect $(a^2 + g^2)b^2$, the direct effect c^2, and the effect b of M on Y. We also derive the variance of the estimated proportion of the genetic variance of Y that is explained by the direct effect c^2 (i.e., the proportion of the additive genetic factor of Y not running through mediator M).

To obtain the variances, we need to compute the (partial) derivatives of the parameters. Deriving the variance of the genetic variance of outcome Y and mediator M is straightforward, as both are defined by a single VC, respectively σ^G_{YY} and σ^G_{MM}. Given the expression of the variance accounted for by the indirect effect as function of the VCs, we have the following partial derivatives for $(a^2 + g^2)b^2$ with respect to the underlying VCs:

$$\frac{\partial(a^2+g^2)b^2}{\partial\sigma^G_{MM}} = \left(\frac{\sigma^E_{MY}}{\sigma^E_{MM}}\right)^2, \tag{57}$$

$$\frac{\partial(a^2+g^2)b^2}{\partial\sigma^E_{MY}} = 2\sigma^G_{MM}\frac{\sigma^E_{MY}}{(\sigma^E_{MM})^2}, \text{ and} \tag{58}$$

$$\frac{\partial(a^2+g^2)b^2}{\partial\sigma^E_{MM}} = -2\sigma^G_{MM}\frac{(\sigma^E_{MY})^2}{(\sigma^E_{MM})^3}. \tag{59}$$

Putting these partial derivatives in gradient vector $\mathbf{g}$ and taking the rows and columns from the variance matrix of the estimated VCs corresponding to σ^G_{MM}, σ^E_{MY}, and σ^E_{MM} respectively, and storing these in 3×3 matrix $\mathbf{V}$, we can now calculate the approximated variance of $(\widehat{a^2+g^2})\widehat{b}^2$ as $\mathbf{g}^\top\mathbf{V}\mathbf{g}$. Accordingly, for the direct effect c^2 we have:

$$c^2 = \sigma^G_{YY} - 2\sigma^G_{MY}\frac{\sigma^E_{MY}}{\sigma^E_{MM}} + \sigma^G_{MM}\left(\frac{\sigma^E_{MY}}{\sigma^E_{MM}}\right)^2 \tag{60}$$

$$\frac{\partial c^2}{\partial\sigma^G_{MM}} = b^2 \tag{61}$$

$$\frac{\partial c^2}{\partial\sigma^G_{MY}} = -2b \tag{62}$$

$$\frac{\partial c^2}{\partial\sigma^G_{YY}} = 1 \tag{63}$$

$$\frac{\partial c^2}{\partial\sigma^E_{MM}} = \frac{2}{\sigma^E_{MM}}\left(\sigma^G_{MY}b - \sigma^G_{MM}b^2\right) \tag{64}$$

$$\frac{\partial c^2}{\partial\sigma^E_{MY}} = \frac{2}{\sigma^E_{MY}}\left(\sigma^G_{MM}b^2 - \sigma^G_{MY}b\right) \tag{65}$$

$$\frac{\partial c^2}{\partial\sigma^E_{YY}} = 0. \tag{66}$$

For the proportion of the genetic variance Y explained by the direct effect c^2 we have:

$$\frac{c^2}{\sigma^G_{YY}} = 1 - 2\frac{\sigma^G_{MY}}{\sigma^G_{YY}}\frac{\sigma^E_{MY}}{\sigma^E_{MM}} + \frac{\sigma^G_{MM}}{\sigma^G_{YY}}\left(\frac{\sigma^E_{MY}}{\sigma^E_{MM}}\right)^2 \tag{67}$$

$$\frac{\partial c^2}{\partial \sigma^G_{MM}} = \frac{b^2}{\sigma^G_{YY}} \tag{68}$$

$$\frac{\partial c^2}{\partial \sigma^G_{MY}} = -\frac{2b}{\sigma^G_{YY}} \tag{69}$$

$$\frac{\partial c^2}{\partial \sigma^G_{YY}} = \frac{2\sigma^G_{MY}b - \sigma^G_{MM}b^2}{(\sigma^G_{YY})^2} \tag{70}$$

$$\frac{\partial c^2}{\partial \sigma^E_{MM}} = \frac{2}{\sigma^E_{MM}\sigma^G_{YY}}\left(\sigma^G_{MY}b - \sigma^G_{MM}b^2\right) \tag{71}$$

$$\frac{\partial c^2}{\partial \sigma^E_{MY}} = \frac{2}{\sigma^E_{MY}\sigma^G_{YY}}\left(\sigma^G_{MM}b^2 - \sigma^G_{MY}b\right) \tag{72}$$

$$\frac{\partial c^2}{\partial \sigma^E_{YY}} = 0. \tag{73}$$

Finally, for b, we obtain:

$$b = \frac{\sigma^E_{MY}}{\sigma^E_{MM}} \tag{74}$$

$$\frac{\partial b}{\partial \sigma^E_{MM}} = -\frac{b}{\sigma^E_{MM}} \tag{75}$$

$$\frac{\partial b}{\partial \sigma^E_{MY}} = \frac{1}{\sigma^E_{MM}}. \tag{76}$$

Section D The idiosyncratic components G^* and E^*

Here, we discuss why the mediation model needs idiosyncratic components G^* and E^*. First, the inclusion of G^* is necessary for the model to be appropriately specified whenever M and Y have an imperfect genetic correlation. In a model without G^*, the genetic component of M can be written as Ga and the genetic component of Y as $G\,(ab + c)$. Thus, Y and M are then both affected only by a single shared genetic factor. Therefore, without G^*, the model would enforce a perfect genetic correlation between M and Y, irrespective of whether there is no mediation, partial mediation, or full mediation. As a consequence, without G^*, any genetic correlation estimate between M and Y that differs significantly from ± 1 would be evidence that the model is misspecified, providing neither an appropriately specified model nor evidence about the degree of mediation.

By including G^*, mediated genetic variance and an imperfect genetic correlation between M and Y can coexist. For example, coefficient a can be relatively small, whereas c, g and b can be of considerable magnitude. In that case, G contributes considerably to the non-mediated genetic variance of Y but not so much to the genetic correlation between Y and M, whereas G^* contributes both to the mediated genetic variance of Y as well as the genetic correlation between M and Y.

It could be argued that it is desirable for the mediation model to include an additional idiosyncratic genetic component, $\widetilde{G}$, that affects only M, but not Y, not even indirectly. However, an additional factor of that nature would violate a core assumption in the mediation framework—in that case, M effectively becomes a composite trait of which one part has effect b on Y whereas another part of M has no effect on Y at all. The mediation framework, however, assumes M has effect b on Y, without making any distinction between different sources of variation in M. Even though the main model in this study does not include the aforementioned additional component $\widetilde{G}$, the model is still sufficiently versatile to incorporate some 'cancelling out'. For example, consider the case where $c = -ab$. In that case, the total effect of component G on Y is zero (i.e., the total effect equals $ab + c = ab - ab = 0$), while its effect on M is still given by a. Thus, our model does permit some of the genetic variance to 'exclusively' affect M, under the constraint that the following assumption is not violated: M has effect b on Y, irrespective of the sources of variance in M.

Finally, our model also permits Y to have idiosyncratic genetic variance that is not shared with M. To illustrate this is indeed possible, consider the case where $a = 0$: G then exclusively affects Y. A more general approach in which Y has its own idiosyncratic genetic factor G' is investigated in Section E.

Regarding E^*, this idiosyncratic factor is necessary for the identification of b. That is, as shown before, b can be written as follows:

$$b = \frac{\sigma^E_{MY}}{\sigma^E_{MM}}. \quad (77)$$

This expression reveals that a non-zero environmental variance of M (after controlling for relevant confounders that might otherwise lead to an omitted variable bias) is needed to estimate the effect of M on Y. Thus, we need an environmental component

for M, which we denote by E^* and which we assign path coefficient f. This component is present (i.e., with $f \neq 0$) in case two conditions are met: (i) M has $h^2_{\text{SNPs}} < 100\%$ even (ii) when controlling for relevant confounders. That is, the residual variance in M is non-zero. Empirically, these are reasonable conditions: h^2_{SNPs} estimates for most traits are well below 50%, even when controlling for a considerable number of potential confounders.

To conclude, with components G^* and E^* we obtain a model that is sufficiently versatile to incorporate various genetic architectures (in terms of h^2_{SNPs} of M and that of Y, and the genetic correlation between M and Y) as well as various degrees of mediation of the genetic variance of Y by M.

Section E An idiosyncratic genetic factor for Y

A drawback of the SEM in Fig A is that Y has no idiosyncratic genetic factor (i.e., a factor which has no bearing on M). This is somewhat different than in typical SEMs, where each observed trait not only has its own idiosyncratic environmental factor (which is the case in our main model, in the form of E^* for M and E for Y), but also its own idiosyncratic genetic factor. In Fig B, we generalize our main model accordingly: this model incorporates an idiosyncratic genetic factor G' for Y, with path coefficient d.

Under this model, the direct effect now also comprises contribution d^2 from idiosyncratic factor G'. Thus, the 'total' direct effect can here be defined as $c^2 + d^2$. Although the SEM in Fig B is statistically indistinguishable from that in Fig A (i.e., both yield the same fit), conceptually they do differ—in case all coefficients are non-zero, the SEM in Fig B assigns to Y an idiosyncratic genetic signal, whereas the SEM in Fig A only permits Y to have genetic signal that is always shared to some degree with M.

Interestingly, if the model in Fig B holds true, MA-GREML still yields consistent estimates of the direct and indirect effect. We show this from two perspectives, *viz*, (i) mapping the SEM in Fig B to the SEM in Fig A with coefficients that yield identical fit and then investigating the direct effect and indirect effect under this identical-fit model, and (ii) evaluating what the direct effect and indirect effect boil down to as function of the VCs under the SEM in Fig B.

Perspective *i*. The fact that the SEMs in Fig B and Fig A are statistically indistinguishable is illustrated by the existence of the following mapping from the coefficients of the SEM in Fig B to those of the SEM in Fig A:

$$c^* = \operatorname{sign}(c)\sqrt{c^2 + d^2}, \tag{78}$$

$$a^* = \frac{ac}{c^*}, \text{ and} \tag{79}$$

$$g^* = \sqrt{g^2 + a^2 - (a^*)^2}. \tag{80}$$

The coefficients for the SEM in Fig A (asterisk notation) yield the same genetic and environmental variance matrix for M and Y as the SEM in Fig B. Yet, again, we stress that the conceptual difference between the two models persists.

If we stick with this latter set of coefficients for the SEM in Fig A (denoted by the asterisks), the direct effect and indirect effect are defined as follows:

$$\text{direct effect} = \left((a^*)^2 + (g^*)^2\right) b^2 \tag{81}$$

$$= \left(\left(\frac{ac}{c^*}\right)^2 + g^2 + a^2 - \left(\frac{ac}{c^*}\right)^2\right) b^2 \tag{82}$$

$$= \left(a^2 + g^2\right) b^2 \tag{83}$$

$$\text{indirect effect} = (c^*)^2 \tag{84}$$

$$= \left(\operatorname{sign}(c)\sqrt{c^2 + d^2}\right)^2 \tag{85}$$

$$= c^2 + d^2. \tag{86}$$

That is, the direct effect and indirect effect defined in terms of the coefficients of the SEM in Fig A boil down to the appropriate functions of coefficients of the SEM in Fig B.

Perspective *ii*. First observe that under the covariance structure implied by the model in Fig B, Eq 39–Eq 44 still hold true, except for Eq 40 which describes how σ^G_{YY} relates to the path coefficients. This equation changes into:

$$\sigma^G_{YY} = \left(a^2 + g^2\right) b^2 + c^2 + d^2 + 2abc. \tag{87}$$

As the equations for σ^E_{MY} and σ^E_{MM} have not changed, their ratio (see Eq 46) still yields a consistent estimator of b. Moreover, as σ^G_{MM} also remains unchanged, the expression for the indirect effect in Eq 56 remains valid.

For the direct effect, we recall from Eq 55 that MA-GREML estimates this direct effect as follows:

$$\text{direct effect} = \sigma^G_{YY} + \sigma^G_{MM}\left(\frac{\sigma^E_{MY}}{\sigma^E_{MM}}\right)^2 - 2\sigma^G_{MY}\frac{\sigma^E_{MY}}{\sigma^E_{MM}}. \tag{88}$$

Substituting the expressions for the VCs in terms of the parameters enables writing the direct effect according to MA-GREML as follows:

$$\begin{aligned}\text{direct effect} &= \left(a^2+g^2\right)b^2 + c^2 + d^2 + 2abc + \left(a^2+g^2\right)b^2 - 2\left(\left(a^2+g^2\right)b + ac\right)b \\ &= 2\left(a^2+g^2\right)b^2 + c^2 + d^2 + 2abc - 2\left(a^2+g^2\right)b^2 - 2abc \\ &= c^2 + d^2.\end{aligned}$$

Thus, indeed, as desired, MA-GREML still provides a consistent estimator of b, the direct effect, and the indirect effect, with the only condition being that the definition of the direct effect has slightly changed: this definition now also includes the contribution of G'. This change is not problematic because the genetic variance contributed by G' indeed is not mediated by M.

To further validate these derivations, we performed simulations using the design described in Section I, but with additional factor G' that only affects Y with weight $d = 1$, and setting all other coefficients also equal to one (i.e., $a = b = c = e = f = g = 1$). Here, the true direct effect equals $c^2 + d^2 = 2$ and the true indirect effect equals $\left(a^2+g^2\right)b^2 = 2$. Across 100 runs, the average estimates are as follows: $\widehat{b} = 0.998$ (SE= 0.014), $\widehat{\text{direct effect}} = 2.012$ (SE= 0.051), and $\widehat{\text{indirect effect}} = 1.989$ (SE= 0.064). These results align very closely with our expectations. Additional parameter estimates averaged across the runs are as follows: $\hat{\sigma}^G_{MM} = 1.998$ (SE= 0.040), $\hat{\sigma}^G_{YY} = 6.011$ (SE= 0.106), $h^2_Y = 0.751$ (SE= 0.006), $h^2_M = 0.667$ (SE= 0.008), $\hat{\rho}^G_{MY} = 0.866$ (SE= 0.004). Here, h^2 denotes SNP-based heritability and ρ^G genetic correlation (with the trait(s) denoted by the subscript(s)). Results per run can be found in Table F in S1 Data.

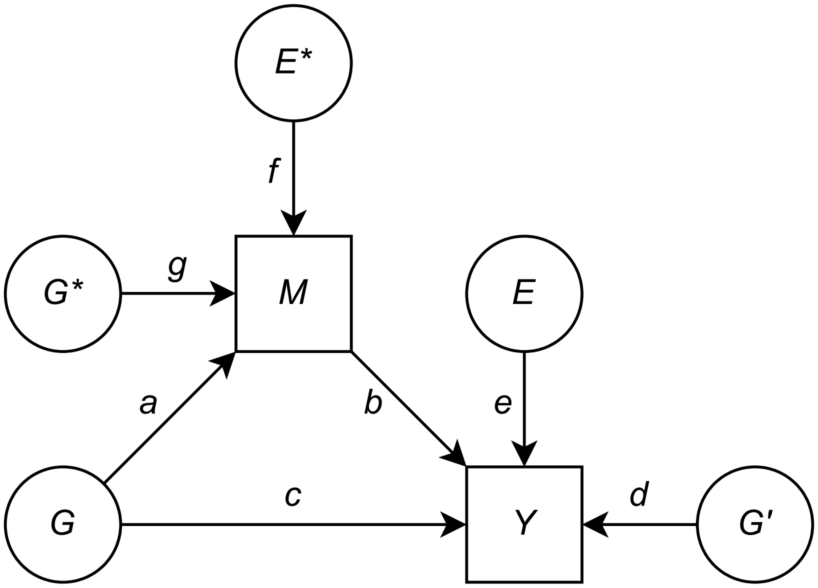

Fig B. Generalized structural equation model (SEM) for mediation analysis using GREML (MA-GREML), to quantify to which extent the genetic component of trait Y affects trait Y through trait M. G, G^*, and G' are latent genetic factors, E and E^* are latent environmental factors, M is the observed mediator, Y is the observed outcome, $(a^2 + g^2)b^2$ is the genetic variance of Y that is mediated by M (*indirect effect*), and $c^2 + d^2$ is the genetic variance of Y that is not mediated by M (*direct effect*); *Full mediation*: direct effect $= 0$ and indirect effect > 0; *Partial mediation*: direct and indirect effect both > 0; *No mediation*: direct effect > 0 and indirect effect $= 0$.

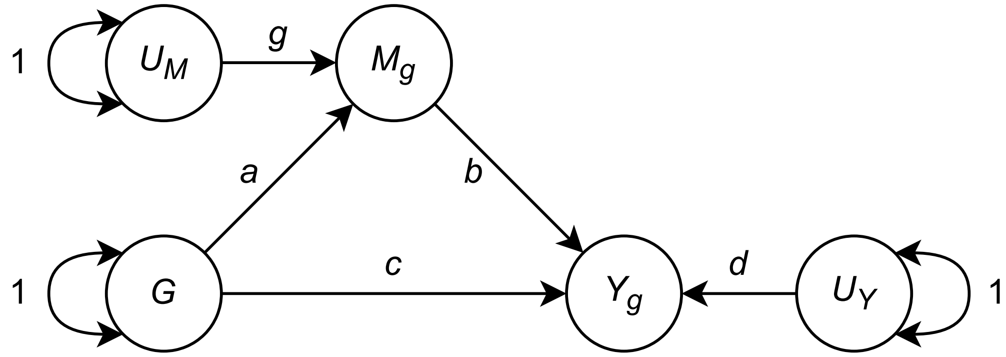

Fig C. Genomic SEM implementation of structural equation model (SEM) shown in Fig B, to quantify to which extent the genetic component of trait Y affects trait Y through trait M. M_g (resp. Y_g) denotes the mediator M (outcome Y) for which we have genome-wide association study summary statistics, U_M (resp. U_Y) denotes an idiosyncratic genetic factor for M (Y), G is a shared genetic factor that directly affects both M and Y.

Section F Mediation analysis using Genomic SEM

Genomic SEM uses genome-wide association study (GWAS) summary statistics to analyze the joint genetic architecture of complex traits. It is a very flexible two-stage structural equation modelling tool that allows users to estimate various models involving forms of mediation [1].

For instance, an online tutorial illustrates how Genomic SEM can be used to assess if ADHD affects income only directly, or whether part of the relation between income and ADHD is mediated by educational attainment. In this example, there are three traits for which GWAS summary statistics are employed.

In the first stage, Genomic SEM estimates the empirical genetic covariance matrix using these summary statistics (e.g., for three traits, effectively three genetic correlations and three heritabilities are estimated). In the second stage, given a user-specified structural equation model, Genomic SEM finds values for the parameters of that model such that (a measure of) the distance between the genetic covariance matrix implied by the model and the empirical genetic covariance matrix is minimized.

Despite its versatility, Genomic SEM is not equipped to answer the question that MA-GREML is able to answer in a bivariate setting, *viz.*, to which degree is the genetic variance of outcome Y mediated by supposed mediator M? The reason for this discrepancy is that although we can – in theory – specify a conceptually similar model in Genomic SEM, such a model is simply not identified within the scope of that method.

This similar model for Genomic SEM is shown in Fig C. Here, shared genetic factor G affects both mediator M and outcome Y (with path coefficients a and c respectively), M has effect b on Y, and both M and Y have an idiosyncratic genetic factor (U_M and U_Y respectively, with path coefficients g and d). In this model, the mediated genetic variance of Y (i.e., the indirect effect) equals $(a^2 + g^2)b^2$ and the non-mediated genetic variance of Y (i.e., the direct effect) equals $c^2 + d^2$. Thus, this model is the 'Genomic SEM implementation' of the model discussed in Section E.

Crucially, however, this model is not identified when applying Genomic SEM, since there are three degrees of freedom (two heritabilities and one genetic correlation) and five parameters (a, b, c, d, g). Even if we omit one of the idiosyncratic factors (e.g., $d = 0$ as in our main model), this model is still not identified when using Genomic SEM.

MA-GREML, on the other hand, identifies b (i.e., the effect of M on Y) using the residual variance of M and the residual covariance between M and Y, and MA-GREML does not attempt to seperate the contributions of a versus g and of c versus d—it only relies on identification of b, $a^2 + g^2$, and $c^2 + d^2$ (in case of the model in Fig B) or c^2 (in case of the model in Fig A). This set-up of MA-GREML leaves sufficient degrees of freedom to identify both the direct and indirect genetic effect. The residual (co)variance can be conceptualised as the phenotypic (co)variance that cannot be explained by the additive random SNP effects and the user-specified control variables with fixed effects.

Even if Genomic SEM would be extended to gain additional degrees of freedom (i.e., using known sample overlap between involved GWASs and resulting estimates of environmental (co)variances) and would employ that information to identify the effect of M on Y (i.e., infer b using the environmental (co)variances), such an approach would then typically yield inconsistent estimates of b and, consequently, inconsistent estimates of the direct and indirect effect. The reason for the inconsistent estimation is that while a well-designed GWAS of M (resp. Y) will control for potential confounders between SNPs and M (Y), neither will – by design – control for confounders of the association between M and Y; that association is simply not what these GWASs are tailored towards.

Section G Mediation analysis using univariate GREML estimation

Traditionally, mediation of the effect of causal factor X on outcome Y via intermediate factor M is assessed using four steps (see e.g., [2]) that can be summarised as follows:

1. Regress Y on X to establish that the cause affects the outcome.
2. Regress M on X to establish that the cause affects the mediator.
3. Regress Y on M to establish that the mediator affects the outcome.
4. Regress Y on X and M.

The coefficient of X in Step 4 can be conceptualised as an estimator of the direct effect and the difference between the coefficient for X in Steps 1 and 4 as an estimator of the

indirect effect. Importantly, in this approach X, M, and Y need to be observed, M needs to precede Y, and other factors that influence both M and Y (i.e., confounders) need to be controlled for.

This approach can be translated to a set of univariate GREML analyses to quantify genetic mediation. Specifically, replacing X by the genetic components of M and Y respectively, the following analogous analyses could be carried out:

1. Perform a univariate GREML analysis of Y, to establish Y has a genetic component G_Y.

2. Perform a univariate GREML analysis of M, to establish M has a genetic component G_M.

3. Regress Y on M to establish the mediator affects the outcome.

4. Perform a univariate GREML analysis of Y including M as fixed-effect covariate.

The genetic variance of Y in Step 4 can now be conceptualised as the direct effect and the difference between the genetic variance of Y in Steps 1 and 4 as an estimator of the indirect effect. However, this approach has an important limitation: under the model in Fig A, G is a classical confounder of the association between M and Y, leading to inconsistent estimation of the effect of M on Y both in Steps 3 and 4. More specifically, the estimator of b (denoted by $\widehat{b}$) in those steps converges to the following quantity:

$$\text{plim}_{N\to\infty}\widehat{b} = b + \frac{ac}{a^2+g^2+f^2}. \tag{89}$$

Thus, univariate GREML can be used to assess mediation in only two extreme scenarios under the model in Fig A, *viz.*, (i) when $a = 0$ or (ii) when there is full mediation (i.e., $c^2 = 0$). In other words, there may be no genetic factor that directly affects both M and Y.

In all other cases, G confounds the inferred association between M and Y in Steps 3 and 4, thus, leading to inconsistent estimation of b, as well as inconsistent estimation of the direct effect and the indirect effect (as the latter two also rely on consistent estimation of b). Finally, even if $ac = 0$, the univariate approach does not provide the means to directly test hypotheses regarding the indirect effect, as that effect is the

difference between separate estimators from Steps 1 and 4, for which we do not know the sampling covariance.

Section H Genetic nurture

Situations where parental factors affect offspring outcomes typically affect estimates from mediation analysis using GREML, unless carefully controlled for. For instance, parental genes may affect educational outcomes beyond the transmitted genes by affecting the rearing environment in which children grow up, a phenomenon known as genetic nurture [3]. Here, we focus on genetic nurture in a specific setting where the genetic nurture components are uncorrelated with the genetic components. To formally introduce genetic nurture in the mediation model, we first need to be slightly more precise about our definition of G and G^*.

Let $\mathbf{x}$ denote the $K \times 1$ vector of standardized genotypes for a given individual and let $\boldsymbol{\beta}$ (resp. $\boldsymbol{\beta}^*$) denote the $K \times 1$ vector of true SNP effects that shape the unobserved genetic factor $\mathbf{G}$ ($\mathbf{G}^*$). That is,

$$G = \mathbf{x}^\top \boldsymbol{\beta}, \text{ and} \tag{90}$$

$$G^* = \mathbf{x}^\top \boldsymbol{\beta}^*. \tag{91}$$

In terms of the standardized genotypes, let X_k denote element $k = 1, \ldots, K$ from $\mathbf{x}$. This element is defined as follows:

$$X_k = \frac{C_k - 2f_k}{\sqrt{2f_k(1 - f_k)}}, \tag{92}$$

where $C_k \in \{0, 1, 2\}$ denotes the coded-allele count (i.e., the raw genotype) and f_k the coded-allele frequency. As genotypes are standardized, G and G^* have mean zero by definition. However, the imposition of unit variance on G and G^* implies that $\boldsymbol{\beta}$ and $\boldsymbol{\beta}^*$ have a certain scale. Yet, this scaling is irrelevant for the considerations at hand.

More important is the fact that G and G^* are not permitted to be correlated. Treating the SNP effects as random, G and G^* are uncorrelated in case $\boldsymbol{\beta}$ and $\boldsymbol{\beta}^*$ are uncorrelated and $\mathbb{E}[\boldsymbol{\beta}] = \mathbb{E}[\boldsymbol{\beta}^*] = \mathbf{0}$, where $\mathbf{0}$ denotes the $K \times 1$ vector of zeros. In that

case, we have:

$$\begin{aligned}
\mathrm{Cov}\left(G, G^*\right) &= \mathbb{E}\left[GG^*\right] && (93)\\
&= \mathbb{E}\left[\mathbf{x}^\top \boldsymbol{\beta} \left(\boldsymbol{\beta}^*\right)^\top \mathbf{x}\right] && (94)\\
&= \mathbb{E}\left[\mathbb{E}\left[\mathbf{x}^\top \boldsymbol{\beta} \left(\boldsymbol{\beta}^*\right)^\top \mathbf{x} \,\middle|\, \mathbf{x}\right]\right] && (95)\\
&= \mathbb{E}\left[\mathbf{x}^\top \mathbb{E}\left[\boldsymbol{\beta} \left(\boldsymbol{\beta}^*\right)^\top \,\middle|\, \mathbf{x}\right] \mathbf{x}\right] && (96)\\
&= \mathbb{E}\left[\mathbf{x}^\top \mathbb{E}\left[\boldsymbol{\beta} \left(\boldsymbol{\beta}^*\right)^\top\right] \mathbf{x}\right] && (97)\\
&= \mathbb{E}\left[\mathbf{x}^\top \mathrm{Cov}\left(\boldsymbol{\beta}, \boldsymbol{\beta}^*\right) \mathbf{x}\right] && (98)\\
&= \mathbb{E}\left[\mathbf{x}^\top \mathbf{0} \mathbf{x}\right] && (99)\\
&= 0. && (100)
\end{aligned}$$

We now have sufficiently precise definitions to also introduce genetic nurture components in the model. We assume there is a parental genetic nurture component that contributes directly towards both M and Y (analogous to G), which we denote by P and that there is a parental genetic nurture component that only contributes to M directly (analogous to G^*), which we denote by P^*. These parental components are weighted sums of parental genotypes, where we assume maternal and paternal genotypes to have the same effects: $\boldsymbol{\gamma}$ to shape P, and $\boldsymbol{\gamma}^*$ to shape P^*. We can now write these two parental genetic nurture components as follows:

$$\begin{aligned}
P &= \mathbf{s}^\top \boldsymbol{\gamma}, \text{ and} && (101)\\
P^* &= \mathbf{s}^\top \boldsymbol{\gamma}^*, && (102)
\end{aligned}$$

where $\mathbf{s}$ is the sum of the maternal and the paternal genotypes of the given individual. Thus, element k of $\mathbf{s}$ is equal to the sum of the standardized maternal genotype and standardized paternal genotype for that SNP k, for $k = 1, \ldots, K$.

Analogous to $\boldsymbol{\beta}$ and $\boldsymbol{\beta}^*$, we assume that $\boldsymbol{\gamma}$ and $\boldsymbol{\gamma}^*$ are uncorrelated random effects. Thus, P and P^* are again uncorrelated. In terms of the downstream effects of these parental genetic nurture components, P has direct effect a_p on M and c_p on Y whereas P^* only has direct effect g_p on M. A graphical representation of this model is shown in Fig B.

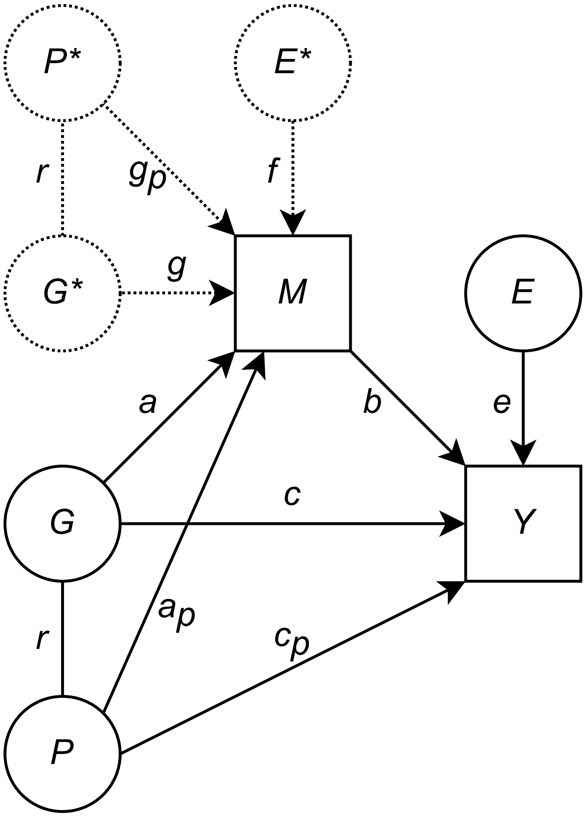

Fig D. Structural equation model (SEM) for mediation analysis using GREML (MA-GREML) including genetic nurture effects. G and G^* are the (latent) genetic components, P and P^* are the (latent) parental genetic components, M is the (observed) mediating factor, E and E^* are the (latent) environmental components, and Y is the (observed) outcome.

Assuming the model in Fig D to be correctly specified, we can now assess how genetic nurture impacts the estimates when the main model in Fig A as implemented in MGREML is used in the empirical analysis. For this comparison, we consider the extreme case that $\text{Cov}(\boldsymbol{\beta}, \boldsymbol{\gamma}) = \mathbf{0}$ and $\text{Cov}(\boldsymbol{\beta}^*, \boldsymbol{\gamma}^*) = \mathbf{0}$, where $\mathbf{0}$ is the $K \times K$ matrix of zeros. This case reflects the scenario where the genetic nurture SNP effects are uncorrelated to the offspring SNP effects. That is, $\text{Cov}(\boldsymbol{\beta}, \boldsymbol{\gamma}) = \text{Cov}(\boldsymbol{\beta}^*, \boldsymbol{\gamma}^*) = \mathbf{0}$. In that case, coefficient r in Fig D equals zero.

Importantly, when GREML is applied in a sample of unrelated individuals it tags about half the genetic nurture variance (provided the genetic nurture SNP effects are uncorrelated with the offspring SNP effects); The other half of the genetic nurture variance shows up as phantom environment variance [4]. Therefore, the implied genetic

and environmental (co)variances as tagged by GREML are as follows:

$$\sigma^G_{MM} = a^2 + g^2 + 0.5a_p^2 + 0.5g_p^2, \tag{103}$$

$$\sigma^G_{YY} = \left(a^2 + g^2 + 0.5a_p^2 + 0.5g_p^2\right) b^2 + c^2 + 0.5c_p^2 + 2abc + a_p b c_p, \tag{104}$$

$$\sigma^G_{MY} = \left(a^2 + g^2 + 0.5a_p^2 + 0.5g_p^2\right) b + ac + 0.5a_p c_p, \tag{105}$$

$$\sigma^E_{MM} = f^2 + 0.5a_p^2 + 0.5g_p^2, \tag{106}$$

$$\sigma^E_{YY} = \left(f^2 + 0.5a_p^2 + 0.5g_p^2\right) b^2 + 0.5c_p^2 + a_p b c_p + e^2, \text{and} \tag{107}$$

$$\sigma^E_{MY} = \left(f^2 + 0.5a_p^2 + 0.5g_p^2\right) b + 0.5a_p c_p. \tag{108}$$

As a result, the expression for b in our main model can be written as follows under the data-generating process in Fig D:

$$b_{main} = \frac{\sigma^E_{MY}}{\sigma^E_{MM}} \tag{109}$$

$$= \frac{\left(f^2 + 0.5a_p^2 + 0.5g_p^2\right) b + 0.5a_p c_p}{f^2 + 0.5a_p^2 + 0.5g_p^2} = b + \frac{a_p c_p}{2f^2 + a_p^2 + g_p^2}. \tag{110}$$

Under our standard assumption that $f \neq 0$, this last expression implies that whenever there is a genetic nurture component that direct affects both M and Y (i.e., $a_p \neq 0$ and $c_p \neq 0$), this leads to inconsistent estimation of b by MA-GREML. That is, as $N \to \infty$ our estimator of b_{main} then equals the true effect of M on Y plus a term that increases in size with a_p and c_p. At its most fundamental level, this is simply an omitted variable bias. This bias can lead to both under- and overestimation of b, and makes our estimator inconsistent. As quantification of the mediated and non-mediated genetic variance of Y hinges crucially on proper identification of b, these derivations suggest that results obtained using MA-GREML can be biased when genetic nurture effects are present.

This is, however, not surprising: the part of the genetic nurture variance that is not tagged by GREML applied to unrelated individuals simply masquerades as environmental variance that affects both M and Y. That is, we have a classical confounder. This insight then immediately points to a solution in the scenario of simple parental genetic nurture: apply the GREML-based mediation analysis to a sample comprising pairs of siblings, and include family-specific dummies as fixed-effect covariates. These family-specific dummies then effectively remove the contribution of

parental genetic nurture to both M and Y. Under this design, GREML-based mediation analysis would give an estimate of the non-mediated and mediated offspring genetic variance (i.e., not considering the nurture variance).

We note that the family-specific dummies approach applied to data on siblings can also deal with other confounders that affect both M and Y (irrespective of whether these are genetically driven or environmentally driven), such as cultural transmission or inter-generational transfers from parent to offspring, provided that these transfers or transmissions are on average shared equally between siblings, thus affecting siblings' expectation for M equally and their expectation for Y equally.

Section I Simulation set-up

Here, we provide more details concerning the simulations described in the main text. We generate R independent datasets (runs). In each run, we generate data for $K = 20,000$ single-nucleotide polymorphisms (SNPs) with minor allele frequency $\geq 5\%$, for a sample comprising $N = 20,000$ unrelated individuals. Using these genetic data, we compute the genomic-relatedness matrix (GRM) needed for MGREML estimation [5] in each run. We then generate, using different parameter settings, our mediator variable M and outcome variable Y based on the structural equation model (SEM) shown in Fig 1 in the main text.

The code for the simulation study is available at the MGREML GitHub page `https://github.com/devlaming/mgreml` in subdirectory *simulations_mediation*. Here, *run.1.limitedmediation.sh* contains Bash code to perform one run of the simulation study and *simulatelimitedmediation.py* contains Python code to simulate data that is called upon by *run.1.limitedmediation.sh*. The general structure of the code is as follows:

1. Generate genetic data for $N = 20,000$ unrelated individuals on $K = 20,000$ SNPs:
   (a) For SNP $k = 1, \ldots, K$:
      i. Draw allele frequency $f_k \sim \text{Beta}(0.35, 0.35)$ and repeat until $f_k \in [0.05, 0.95]$.
      ii. For individual $j = 1, \ldots, N$: draw genotype $G_{jk} \sim Binom(2, f_k)$.
      iii. Compute empirical allele frequency: $k_j = (G_{1j} + \ldots + G_{Nj})[2N]^{-1}$.

iv. For individual $j = 1, \ldots, N$: standardize genotype:

$X_{jk} = (G_{jk} - 2k_j)[2k_j(1 - k_j)]^{-0.5}$.

(b) Let $\mathbf{X}$ denote the resulting $N \times K$ matrix of standardized genotypes.

(c) Compute $\mathbf{A} = K^{-1}\mathbf{X}\mathbf{X}^\top$ and store this GRM in binary format.

2. Generate genetic factors and environmental effects, with no covariance between these factors, conditional on the genotypes:

   (a) For the genetic factor $t = 1, 2$:

      i. Draw SNP effects: $\boldsymbol{\beta}_t \sim \mathcal{N}(\mathbf{0}, \mathbf{I}_K)$.

      ii. Compute genetic factor: $\mathbf{g}_t^* = \mathbf{X}\boldsymbol{\beta}_t$.

      iii. Standardize $\mathbf{g}_t^*$ to have empirical mean zero and unit variance $\rightarrow \mathbf{g}_t$.

   (b) For the environmental factor of trait $t = 1, 2$:

      i. Draw environmental effects: $\boldsymbol{\varepsilon}_t^* \sim \mathcal{N}(\mathbf{0}, \mathbf{I}_N)$.

      ii. Standardize $\boldsymbol{\varepsilon}_t^*$ to have empirical mean zero and unit variance $\rightarrow \boldsymbol{\varepsilon}_t$.

3. Generate confounders (to mimic a more realistic setting), with the first confounder being an intercept:

   (a) For Confounder 1:

      i. Set $\mathbf{c}_1$ to a vector of length N containing 1's only.

   (b) For Confounder $k = 2, \ldots, 10$:

      i. Generate a vector of length N as $\mathbf{c}_k \sim \mathcal{N}(\mathbf{0}, \mathbf{I}_N)$.

   (c) Let $\mathbf{C}$ denote the resulting $N \times 10$ matrix of confounders.

4. Draw the effects of the confounders on the mediator M and the outcome Y:

   (a) The effects of the confounders on the mediator $\mathbf{d}_M \sim \mathcal{N}(\mathbf{0}, \mathbf{I}_k)$.

   (b) The effects of the confounders on the outcome $\mathbf{d}_Y \sim \mathcal{N}(\mathbf{0}, \mathbf{I}_k)$.

5. Set the parameters in the SEM (Fig 1 in the main text):

   (a) Set e and f to 1.

   (b) Set a, b, c, and g according to the different simulation scenarios (see below).

6. Generate the mediator M and the outcome Y:

   (a) $\mathbf{m} = \mathbf{C}\mathbf{d}_M + \mathbf{g}_1 a + \mathbf{g}_2 g + \boldsymbol{\varepsilon}_2 f$.

   (b) $\mathbf{y} = \mathbf{C}\mathbf{d}_Y + \mathbf{m} b + \mathbf{g}_1 c + \boldsymbol{\varepsilon}_1 e$.

7. Use the binary GRM and simulated mediator M and outcome Y to perform the mediation analysis using MGREML.

We analyze the estimated parameters and test statistics in a baseline scenario with partial mediation and four additional scenarios involving no mediation and full mediation. The values for a, b, c, and g for the different scenarios are described below. For each scenario, we perform $R = 100$ runs.

***Baseline*: Partial mediation.** Here, we set $a = b = c = g = 1$.

Scenario (*i*): No mediation, where M has no genetic variance but M does have an effect on Y. Here, we set $a = g = 0$ and $b = c = 1$.

Scenario (*ii*): No mediation, where M has genetic variance but M does not have an effect on Y. Here, we set $b = 0$ and $a = c = g = 1$.

Scenario (*iii*): No mediation, where M has no genetic variance and M does not have an effect on Y. Here, we set $a = b = g = 0$ and $c = 1$.

Scenario (*iv*): Full mediation. Here, we set $a = b = g = 1$ and $c = 0$.

Section J Extended simulation results

In the main text we provide the most relevant output of the simulations described in Section I: The estimated effect of mediator M on outcome Y (i.e., $\widehat{b}$), the estimated direct effect (i.e., $\widehat{c^2}$), and the estimated indirect effect (i.e, $\widehat{(a^2+g^2)}\widehat{b^2}$). Here, in Table A, we provide the average estimates of the genetic variances of mediator M and outcome Y, h^2_{SNPs} of M and Y, and the genetic correlation between M and Y.

All results are in line with expectations. In all scenarios, the genetic variance of both the mediator M and the outcome Y are precisely estimated with small standard errors. h^2_{SNPs} of both the mediator M and the outcome Y are quite precisely estimated, although in the scenarios where the mediator is not heritable (Scenarios (i) and (iii)), we observe that h^2_{SNPs} estimates are marginally above zero. This is not surprising for estimators of quantities that lie on the edge of the parameter case (e.g., observe that

Scenario	Parameter settings	$\hat{\sigma}^G_{MM}$	$\hat{\sigma}^G_{YY}$	h^2_Y	h^2_M	$\hat{\rho}^G_{MY}$
Baseline	$b = 1,\, c = 1,\, (a^2 + g^2) = 2$	2.000	4.997	0.714	0.667	0.949
		(0.040)	(0.093)	(0.007)	(0.008)	(0.002)
(i)	$b = 1,\, c = 1,\, (a^2 + g^2) = 0$	0.003	1.005	0.335	0.003	0.193
		(0.010)	(0.036)	(0.011)	(0.010)	(5546)
(ii)	$b = 0\ ,\, c = 1,\, (a^2 + g^2) = 2$	2.000	0.998	0.499	0.667	0.707
		(0.040)	(0.025)	(0.009)	(0.008)	(0.012)
(iii)	$b = 0,\, c = 1,\, (a^2 + g^2) = 0$	0.003	0.999	0.499	0.003	0.146
		(0.010)	(0.026)	(0.010)	(0.010)	(2601)
(iv)	$b = 1,\, c = 0,\, (a^2 + g^2) = 2$	2.001	2.006	0.502	0.667	0.999
		(0.040)	(0.050)	(0.009)	(0.008)	(0.003)

Table A. Average estimates and corresponding standard errors of estimated parameters in the mediation model (100 simulation runs for each scenario). $\hat{\sigma}^G_{MM}$ is the estimated genetic variance of mediator M; $\hat{\sigma}^G_{YY}$ is the estimated genetic variance of outcome Y; h^2_M is the estimated SNP-based heritability of M; h^2_Y is the estimated SNP-based heritability of Y; $\hat{\rho}^G_{MY}$ is the estimated genetic correlation between M and Y; Average standard errors in parentheses.

$h^2_{\text{SNPs}} \in [0, 1]$)—unbiasedness is not the property one should focus on here; the focus lies on consistency (i.e., do the estimators converge to the true parameters as $N \to \infty$). For the genetic correlation between the mediator and the outcome, we observe that in Scenario (i) and (iii) there are very high standard errors. In these scenarios there is no genetic variance in the mediator and, hence, the genetic correlation estimate is extremely imprecise which is reflected by the high standard errors. In fact, in terms of the true parameters, the true genetic correlation is not even defined in Scenarios (i) and (iii), as the true genetic correlation then involves a division by zero.

Section K Partitioning of the GRM by functional categories

Here we provide additional empirical results for the four later-life outcomes analyzed in the main text with educational attainment as mediator. As a first step, we used functional annotations as provided in the data file `https://data.broadinstitute.org/alkesgroup/LDSCORE/1000G_Phase1_cell_type_groups.tgz` to select SNPs for belonging to cell type groups Adrenal/Pancreas (69,744 SNPs), Cardiovascular (90,500 SNPs), Cental Nervous System (10,773 SNPs), Connective/Bone (82,771 SNPs), Gastrointestinal (122,734 SNPs), Immune/Hematopoietic (152,417 SNPs), Kidney (32,030 SNPs), Liver (51,847 SNPs), Muscle/Skeletal (80,803 SNPs), and Other (143,634

SNPs). Using the relevant SNPs, we construct 10 genomic relationship matrices (GRMs).

For most functional categories, we find nominally significant (p-value < 0.05) indirect effects through educational attainment. The most notable exceptions are Kidney and Liver: For these functional categories the indirect effects are insignificant for all four later-life health outcomes. We caution that MGREML [6] cannot fit multiple GRMs in the model simultaneously. Therefore, the estimated mediated and non-mediated genetic variance for a given GRM (e.g., one functional category) may still be influenced by similar GRMs (e.g., representing other functional categories).

Functional category	Body Mass Index	Cognition	Mental health	Self-reported health
Adrenal/Pancreas	0.00641	0.06650	0.01854	0.01301
Cardiovascular	0.02982	0.03427	0.00063	0.03974
Cental Nervous System	0.00414	0.00183	0.00002	0.00001
Connective/Bone	0.02912	0.28329	0.01522	0.05309
Gastrointestinal	0.00001	0.00374	0.00006	0.00019
Immune/Hematopoietic	0.00030	0.03268	0.00013	<0.00001
Kidney	0.53361	0.98649	0.21668	0.87976
Liver	0.82418	0.96891	0.16783	0.92279
Muscle/Skeletal	0.01067	0.00993	0.00313	0.00004
Other	0.00062	0.00451	0.00022	0.00001
All	0.02534	0.01101	0.18229	0.01001

Table B. Evidence (p-values) regarding whether the genetic components of the four later-life health outcomes affect the later-life health outcomes through educational attainment. Genomic relationship matrices are constructed using SNPs in the specified functional category.

References

1. Grotzinger AD, Rhemtulla M, De Vlaming R, Ritchie SJ, Mallard TT, Hill WD, et al. Genomic structural equation modelling provides insights into the multivariate genetic architecture of complex traits. Nat Human Beh. 2019; 3(5):513-525.

2. Baron RM, Kenny DA. The moderator–mediator variable distinction in social psychological research: Conceptual, strategic, and statistical considerations. J Pers Soc Psychol. 1986;51(6):1173–1182.

3. Kong A, Thorleifsson G, Frigge ML, Vilhjálmsson BJ, Young AI, Thorgeirsson TE, et al. The nature of nurture: Effects of parental genotypes. Science. 2018;359(6374):424–428.

4. Van Kippersluis H, Biroli P, Galama TJ, Von Hinke S, Meddens SFW, Muslimova D, et al. Overcoming attenuation bias in regressions using polygenic indices: A comparison of approaches. BioRxiv. 2021.

5. De Vlaming R, Slob EAW, Jansen PR, Dagher A, Koellinger PD, Groenen PJF, Rietveld CA. Multivariate analysis reveals shared genetic architecture of brain morphology and human behavior. Comm Biol. 2021;4(1):1180.

6. De Vlaming R, Slob EAW. MGREML v1.0.0. doi:10.5281/zenodo.5499768. 2021.
